# Nature-Inspired *O*-Benzyl Oxime-Based Derivatives as New Dual-Acting Agents Targeting Aldose Reductase and Oxidative Stress

**DOI:** 10.3390/biom12030448

**Published:** 2022-03-14

**Authors:** Lidia Ciccone, Giovanni Petrarolo, Francesca Barsuglia, Carole Fruchart-Gaillard, Evelyne Cassar Lajeunesse, Adeniyi T. Adewumi, Mahmoud E. S. Soliman, Concettina La Motta, Elisabetta Orlandini, Susanna Nencetti

**Affiliations:** 1 Department of Pharmacy, University of Pisa, Via Bonanno 6, 56126 Pisa, Italy; lidia.ciccone@unipi.it (L.C.); giovanni.petrarolo@phd.unipi.it (G.P.); f.barsuglia@studenti.unipi.it (F.B.); 2 Département Médicaments et Technologies pour la Santé (DMTS), Université Paris Saclay, Commissariat à l’Énergie Atomique et aux Énergies Alternatives (CEA), Institut National de Recherche pour l’Agricolture, l’Alimentation et l’Environment (INRAE), SIMoS, 91191 Gif-sur-Yvette, France; carole.fruchart@cea.fr (C.F.-G.); evelyne.cassar@cea.fr (E.C.L.); 3 Centre for Instrumentation Sharing, University of Pisa (CISUP), Lungarno Pacinotti 43, 56126 Pisa, Italy; elisabetta.orlandini@unipi.it; 4 Molecular Bio-Computation and Drug Design Laboratory, School of Health Science, Westville Campus, University of KwaZulu-Natal, Durban 4001, South Africa; jesutomisin0707@gmail.com (A.T.A.); soliman@ukzn.ac.za (M.E.S.S.); 5 Department of Earth Sciences, University of Pisa, Via Santa Maria 53, 56126 Pisa, Italy; 6 Research Center “E. Piaggio”, University of Pisa, Largo Lucio Lazzarino 1, 56122 Pisa, Italy

**Keywords:** aldose reductase, aldose reductase inhibitors, anti-oxidants, benzaldehyde *O*-benzyl oximes, multifunctional compounds

## Abstract

Aldose reductase (ALR2) is the enzyme in charge of developing cellular toxicity caused by diabetic hyperglycemia, which in turn leads to the generation of reactive oxygen species triggering oxidative stress. Therefore, inhibiting ALR2 while pursuing a concomitant anti-oxidant activity through dual-acting agents is now recognized as the gold standard treatment for preventing or at least delaying the progression of diabetic complications. Herein we describe a novel series of (*E*)-benzaldehyde *O*-benzyl oximes **6a–e**, **7a–e**, **8a–e**, and **9–11** as ALR2 inhibitors endowed with anti-oxidant properties. Inspired by the natural products, the synthesized derivatives are characterized by a different polyhydroxy substitution pattern on their benzaldehyde fragment, which proved crucial for both the enzyme inhibitory activity and the anti-oxidant capacity. Derivatives (*E*)-2,3,4-trihydroxybenzaldehyde *O*-(3-methoxybenzyl) oxime (**7b**) and (*E*)-2,3,4-trihydroxybenzaldehyde *O*-(4-methoxybenzyl) oxime (**8b**) turned out to be the most effective dual-acting products, proving to combine the best ALR2 inhibitory properties with significant anti-oxidant efficacy.

## 1. Introduction

Aldose reductase (alditol:NADP^+^ oxidoreductase, EC 1.1.1.21, AKR1B1, ALR2) is commonly acknowledged as the enzyme in charge of the development of long-term diabetic complications. Therefore, its inhibition represents the key tool to prevent or at least delay the progression and severity of the pathological changes that diabetics face over time [1].

**Figure 1:**
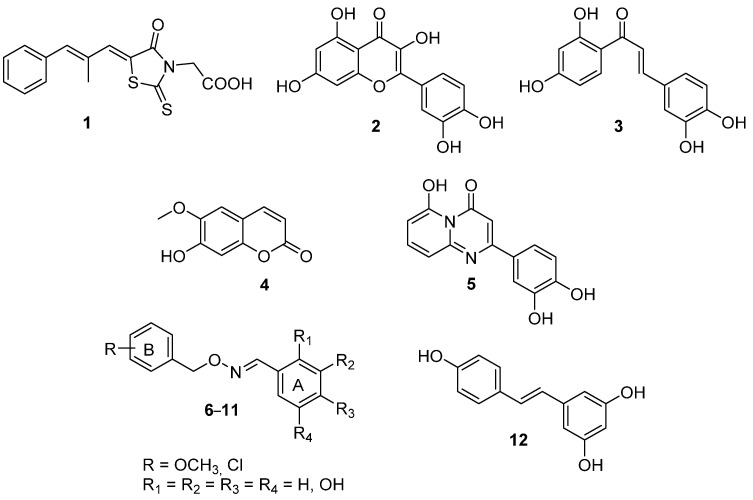
Representative examples of ARIs.

This consideration has prompted medicinal chemists to design many structurally different compounds as potent ALR2 inhibitors (ARIs), with a goal to develop a gold standard treatment for diabetic diseases affecting the heart, nerve, eye, and kidney. However, a true and widespread ARI therapy has not yet become a reality despite expectations. Epalrestat (**1**, Figure 1) is the only drug successfully marketed in Japan, India, and China to treat diabetic patients with neuropathy. Most of the compounds under investigation displayed a limited clinical efficacy, often due to unfavorable pharmacokinetic properties or even unexpected toxicities. Accordingly, the obtainment of effective and safe ARIs is still an unmet research goal deserving to be continued.

A thorough survey of the most recent literature in the ARI field highlights an increasingly interest in natural compounds, mainly represented by polyhydroxylated flavonoids as quercetin (**2**, Figure 1), chalcones as butein (**3**, Figure 1), and coumarins as scopoletin (**4**, Figure 1), obtained from different plant species [2]. Besides fully addressing the current policy of the World Health Organization (WHO), which promotes the integration of traditional herbal medicines into the health care practices of countries [3], the use of these derivatives is justified by their multifunctional profile, which combines a promising ARI activity with a potent anti-oxidant efficacy. This latter aspect, in particular, is emerging as highly desirable in the setting up of novel ARIs. In fact, diabetes is a pathology characterized by both an increased formation of free radicals and a decreased antioxidant capacity, and the resulting oxidative stress plays a crucial role in the onset and progression of cell damage. Moreover, it may also trigger post-translational modification of key proteins, including ALR2, whose oxidation of the CYS299 residue leads to a marked reduction in sensitivity to ARIs, thus compromising the therapeutic effectiveness of these compounds [4,5].

Despite their biological versatility, polyhydroxylated natural derivatives generally struggle to become therapeutic agents, as most of them show low selectivity for the desired target and poor pharmacokinetic and pharmacodynamic properties. Therefore, instead of being claimed as active ingredients *per se*, these compounds should be better utilized as inspiring scaffolds by medicinal chemists, who have the tools to improve their functional profile through suitable structural modifications [6].

Pursuing our interest in the ARI field [7,8,9,10,11,12] and prompted by our successful experience of turning the flavonoid core into the bioisosteric pyrido[1,2-*a*]pyrimidin-4-one nucleus, by obtaining potent and selective anti-oxidant ARIs such as **5** (Figure 1) [13], we developed a novel series of polyhydroxylated (*E*)-benzaldehyde *O*-benzyl oximes **6–11** (Figure 1) as prototypes of dual-acting ARIs inspired by the stilbene derivative resveratrol (**12**, Figure 1), a natural compound overlooked by the ARI community. Different polyhydroxylated substitution patterns were explored on the A phenyl ring of the compounds (Figure 1), being these groups decisive for the anti-oxidant activity and, possibly, for the interaction with the catalytic binding site of the enzyme, as demonstrated for selected natural derivatives [13]. In addition, methoxy residues and chlorine atoms were inserted on the B phenyl ring (Figure 1), as they are reported to increase the lipophilicity of ARIs improving their penetration through membranes and tissues, including the blood–brain barrier [14].

The novel compounds were investigated in vitro for their capacity to inhibit the human recombinant ALR2 and their anti-oxidant properties. To this end, both the TBARS and the DPPH assays were exploited to account for the ability of the compounds to protect from lipid peroxidation, which is significantly increased in diabetes even in well-controlled patients, and to scavenge free radicals, generated in a significant amount by the hyperglycemic conditions [15,16]. Moreover, docking simulations and molecular dynamic studies of the most active derivatives into the human ALR2 binding site were also carried out to rationalize the structure–activity relationships (SARs) observed.

## 2. Materials and Methods

### 2.1. Chemistry

Analytical grade reagents and solvents were purchased from Sigma–Aldrich (St. Louis, MO, USA), and were used as supplied. ^1^H-NMR spectra were determined with a Bruker Ultrashield^TM^ 400 MHz (Fällander, Switzerland). The following abbreviations are being used in this paper: singlet (s), doublet (d), triplet (t), doublet of doublets (dd), doublet of triplet (dt), and multiplet (m). Melting points (m.p.) were measured with a Leica Galen III microscope (Leica/Cambridge Instruments) and were uncorrected. Reactions were monitored by thin-layer chromatography (TLC) on silica gel plates containing a fluorescent indicator (Merck Silica Gel 60 F254); spots were detected under UV light (254 nm). Na_2_SO_4_ was always used as the drying agent. Evaporation was carried out under reduced pressure (rotating evaporator). IR spectra of the synthesized compounds were recorded using a Cary 600 Series FTIR Spectrometer, (Agilent Technologies, Mulgrave, Victoria, Australia). The purity of the target inhibitors was determined by HPLC analysis, using a Shimadzu LC-20AD liquid chromatograph (PDA, 250–500 nm) and a Luna C18 column (250 mm × 4.6 mm, 5 μm, Phenomenex), with a gradient of 100% methanol and a flow rate of 0.2 mL/min. All the compounds showed percent purity values ≥95%.

#### General Procedure for the Synthesis of (E)-Polyhydroxy Substituted Benzaldehyde O-(benzyl) Oximes **6a–e**, **7a–e**, **8a–e**, and **9–11**

To a solution of the commercially available polyhydroxybenzaldehyde **17–21** (0.264 mmol) in MeOH (4 mL), an aqueous solution (1 mL) of the opportune hydroxylamine hydrochloride **16a–e** (0.264 mmol) [17,18,19] was added. The reaction mixture was stirred for 1 h at room temperature until the disappearance of the starting material (TLC analysis). The mixture was evaporated to dryness and the resulting crude was added with water and extracted with EtOAc. The organic phase was washed (3x) with water, dried (Na_2_SO_4_), filtered, and evaporated under reduced pressure to obtain a crude solid.

(*E*)-3,5-Dihydroxybenzaldehyde *O*-(2-methoxybenzyl) oxime, **6a**.

Compound **6a** was obtained starting from the 3,5-dihydroxybenzaldehyde **17**, following the general procedure. The crude was purified by crystallization (CHCl_3_/hexane) affording compound **6a** as a yellow solid. Yield 40%. M.p.: 55–57 °C. ^1^H-NMR (400 Hz; DMSO-*d*_6_) δ: 9.41 (s, 2H, -OH); 8.09 (s, 1H, -CH=N-); 7.32 (t, 2H, *J* = 7.60 Hz, Ar-H); 7.04-7.02 (m, 1H, Ar-H); 6.95 (t, 1H, *J* = 7.60 Hz, Ar-H); 6.44 (d, 2H, *J* = 1.88 Hz, Ar-H); 6.24 (t, 1H, *J* = 1.88 Hz, Ar-H); 5.12 (s, 2H, -OCH_2_-); 3.82 (s, 3H, OCH_3_). ^13^C-NMR (100 Hz; DMSO-*d*_6_) δ: 158.6, 157.0, 149.2, 133.5, 129.6, 129.3, 125.3, 120.1, 110.8, 105.0, 104.2, 70.4, 55.4.

(*E*)-3,4,5-Trihydroxybenzaldehyde *O*-(2-methoxybenzyl) oxime, **6b**.

Compound **6b** was obtained starting from the 2,3,4-trihydroxybenzaldehyde **18**, following the general procedure. The crude was purified by trituration with hexane affording compound **6b** as a yellow solid. Yield 55%. M.p.: 79–82 °C. ^1^H-NMR (400 MHz; DMSO-*d*_6_) δ: 9.44 (s, 2H, -OH); 8.45 (s, 1H, -OH); 8.29 (s, 1H, -CH=N-); 7.34-7.30 (m, 2H, Ar-H); 7.02 (d, 1H, *J* = 8.00 Hz, Ar-H); 6.95 (dt, 1H, *J*_1_ = 8.0 Hz, *J*_2_ = 1.0 Hz, Ar-H); 6.75 (d, 1H, *J* = 8.52 Hz, Ar-H); 6.32 (d, 1H, *J* = 8.52 Hz, Ar-H); 5.09 (s, 2H, -OCH_2_-); 3.81 (s, 3H, -OCH_3_). ^13^C-NMR (100 Hz; DMSO-*d*_6_) δ: 158.6, 157.3, 149.6, 148.4, 146.2, 132.8, 130.1, 129.5, 125.3, 120.2, 119.5, 111.0, 109.7, 107.8, 70.4, 55.5.

(*E*)-3,4-Dihydroxybenzaldehyde *O*-(2-methoxybenzyl) oxime, **6c**.

Compound **6c** was obtained starting from the 3,4-dihydroxybenzaldehyde **19**, following the general procedure. The crude was purified by trituration with hexane affording compound **6c** as a yellow solid. Yield 80%. M.p.: 88–90 °C. ^1^H-NMR (400 MHz; DMSO-*d*_6_) δ: 9.33 (s, 1H, -OH); 9.16 (s, 1H, -OH); 8.07 (s, 1H, -CH=N-); 7.33-7.28 (m, 2H, Ar-H); 7.03 (dd, 2H, *J*_1_ = 8.16 Hz, *J*_2_ = 1.96 Hz, Ar-H); 6.93 (dt, 1H, *J*_1_ = 8.16 Hz, *J*_2_ = 1.0 Hz, Ar-H); 6.83 (dd, 1H, *J*_1_ = 8.16 Hz, *J*_2_ = 1.96 Hz, Ar-H); 6.73 (d, 1H, *J* = 8.16 Hz, Ar-H); 5.08 (s, 2H, -OCH_2_-); 3.81 (s, 3H, -OCH_3_). ^13^C-NMR (100 Hz; DMSO-*d*_6_) δ: 156.9, 148.9, 147.6, 145.5, 126.5, 129.1, 125.5, 123.2, 119.9, 115.5, 112.8, 110.7, 109.7, 70.2, 55.3.

(*E*)-2,5-Dihydroxybenzaldehyde *O*-(2-methoxybenzyl) oxime, **6d**.

Compound **6d** was obtained starting from the 2,5-dihydroxybenzaldehyde **20**, following the general procedure. The crude was purified by trituration with hexane affording compound **6d** as a yellow solid. Yield 75%. M.p.: 104–106 °C. ^1^H-NMR (400 Hz; DMSO-*d*_6_) δ: 9.27 (s, 1H, -OH); 8.91 (s, 1H, -OH); 8.33 (s, 1H, -CH=N-); 7.32 (dd, 2H, *J*_1_ = 8.0 Hz, *J*_2_ = 1.2 Hz, Ar-H); 7.02 (d, 1H, *J*_1_ = 8.0 Hz, Ar-H); 6.94 (dt, 1H, *J*_1_ = 7.6 Hz, *J*_2_ = 1.2 Hz, Ar-H); 6.89 (d, 1H, *J* = 2.40 Hz, Ar-H); 6.68 (dt, 2H, *J*_1_ = 12 Hz, *J*_2_ = 2.40, Hz Ar-H); 5.13 (s, 2H, -OCH_2_); 3.81 (s, 3H, -OCH_3_). ^13^C-NMR (100 Hz; DMSO-*d*_6_) δ: 157.1, 149.8, 148.9, 147.0, 129.8, 129.4, 125.2, 120.2, 118.7, 117.6, 117.0, 112.3, 110.8, 70.5, 55.4.

(*E*)-2,4-Dihydroxybenzaldehyde *O*-(2-methoxybenzyl) oxime, **6e**.

Compound **6e** was obtained starting from the 2,4-dihydroxybenzaldehyde **21**, following the general procedure. The crude was purified by trituration with hexane affording compound **6e** as a yellow solid. Yield 75%. M.p.: 123–125 °C. ^1^H-NMR (400 Hz; DMSO-*d*_6_) δ: 9.87 (s, 1H, -OH); 9.83 (s, 1H, -OH); 8.29 (s, 1H, -CH=N-); 7.34–7.25 (m, 3H, Ar-H); 7.01 (d, 1H, *J* = 8.0 Hz, Ar-H); 6.95 (dt, 1H, *J*_1_ = 8.0 Hz, *J*_2_ = 0.8 Hz, Ar-H); 6.29–6.26 (m, 2H, Ar-H); 5.08 (s, 2H, -OCH_2_-); 3.81 (s, 3H, -OCH_3_). ^13^C-NMR (100 Hz; DMSO-*d*_6_) δ: 160.3, 157.8, 157.1, 148.0, 129.9, 129.4, 125.3, 120.1, 110.8, 108.9, 107.7, 102.4, 70.2, 55.4.

(*E*)-3,5-Dihydroxybenzaldehyde *O*-(3-methoxybenzyl) oxime, **7a**.

Compound **7a** was obtained starting from the 3,5-dihydroxybenzaldehyde **17**, following the general procedure. The crude was purified by trituration with hexane affording compound **7a** as a yellow solid. Yield 64%. M.p.: 44–47 °C. ^1^H-NMR (400 Hz; DMSO-*d*_6_) δ: 9.43 (s, 2H, -OH), 8.10 (s, 1H, -CH=N-); 7.28 (t, 1H, *J*_1_ = 7.0 Hz, Ar-H); 6.94 (dd, 1H, *J*_1_ = 7.0 Hz, *J*_2_ = 1.20 Hz, Ar-H); 6.98–6.86 (m, 2H, Ar-H); 6.46 (d, 2H, *J* = 2.20 Hz, Ar-H); 6.24 (t, 1H, *J* = 2.20 Hz, Ar-H); 5.09 (s, 2H, -OCH_2_-); 3.75 (s, 3H, -OCH_3_). ^13^C-NMR (100 Hz; DMSO-*d*_6_) δ: 159.2, 158.6, 149.9, 139.3, 133.4, 129.4, 120.2, 113.5, 113.1, 105.0, 104.3, 75.1, 55.0.

(*E*)-3,4,5-Trihydroxybenzaldehyde *O*-(3-methoxybenzyl) oxime, **7b**.

Compound **7b** was obtained starting from the 2,3,4-trihydroxybenzaldehyde **17**, following the general procedure. The crude was purified by trituration with CHCl_3_, affording compound **7b** as a yellow solid. Yield 86%. M.p.: 90–92 °C. ^1^H-NMR (400 MHz; DMSO-*d*_6_) δ: 9.48 (s, 1H, -OH); 9.35 (s, 1H, -OH); 8.46 (s, 1H, -OH); 8.32 (s, 1H, -CH=N-); 7.28 (t, 1H, *J* = 8.0 Hz, Ar-H); 6.95 (dd, 2H, *J*_1_ = 8.0 Hz, *J*_2_ = 1.44 Hz, Ar-H); 6.89-6.87 (m, 1H, Ar-H); 6.78 (d, 1H, *J* = 8.52 Hz, Ar-H); 6.34 (d, 1H, *J* = 8.52 Hz, Ar-H); 5.08 (s, 2H, -OCH_2_-); 3,75 (s, 3H, -OCH_3_). ^13^C-NMR (100 Hz; DMSO-*d*_6_) δ: 159.2, 149.5, 148.4, 146.1, 139.2, 132.8, 129.4, 120.3, 119.2, 113.7, 113.2, 109.6, 107.8, 75.0, 55.0.

(*E*)-3,4-Dihydroxybenzaldehyde *O*-(3-methoxybenzyl) oxime, **7c**.

Compound **7c** was obtained starting from the 3,4-dihydroxybenzaldehyde **19**, following the general procedure. The crude was purified by trituration with hexane affording compound **7c** as a yellow solid. Yield 54%. M.p. 90-92 °C. ^1^H-NMR (400 Hz; DMSO-*d*_6_) δ: 9.33 (br, 2H, -OH); 8.07 (s, 1H, -CH=N-); 7.28–7.24 (m, 2H, Ar-H); 7.04 (dd, 2H, *J*_1_ = 8.16 Hz, *J*_2_ = 1.96 Hz, Ar-H); 6.95–6.93 (m, 1H, Ar-H); 6.85 (dt, 1H, *J*_1_ = 8.16 Hz, *J*_2_ = 1.96 Hz, Ar-H); 6.73 (d, 1H, *J*_1_ = 8.16 Hz, Ar-H); 5.05 (s, 2H, -OCH_2_-); 3.74 (s, 3H, -OCH_3_). ^13^C-NMR (100 Hz; DMSO-*d*_6_) δ: 159.8, 144.0, 147.6, 122.0, 120.8, 115.4, 113.9, 113.7, 113.0, 117.6, 117.0, 112.3, 110.8, 70.5, 55.4.

(*E*)-2,5-Dihydroxybenzaldehyde *O*-(3-methoxybenzyl) oxime, **7d**.

Compound **7d** was obtained starting from the 2,5-dihydroxybenzaldehyde **20**, following the general procedure. The crude was purified by trituration with hexane affording compound **7d** as a yellow solid. Yield 55%. M.p.: 73–75 °C. ^1^H-NMR (400 Hz; DMSO-*d*_6_) δ: 9.27 (s, 1H, -OH); 8.92 (s, 1H, -OH); 8.36 (s, 1H, -CH=N-); 7.32 (t, 1H, *J* = 8.0 Hz, Ar-H); 6.92 (dd, 2H, *J*_1_ = 8.0 Hz, *J*_2_ = 1.6 Hz, Ar-H); 6.91 (d, 1H, *J* = 2.8, Ar-H); 6.89–6.80 (m, 1H, Ar-H); 6.67 (dt, 2H, *J*_1_ = 12.0 Hz, *J*_2_ = 2.8 Hz, Ar-H); 5.12 (s, 2H, -OCH_2_-); 3.76 (s, 3H, -OCH_3_). ^13^C-NMR (100 Hz; DMSO-*d*_6_) δ: 159.2, 149.9, 148.9, 146.8, 139.2, 129.4, 120.2, 118.8, 117.6, 117.0, 113.6, 113.2, 112.0, 75.1, 55.0.

(*E*)-2,4-Dihydroxybenzaldehyde *O*-(2-methoxybenzyl) oxime, **7e**.

Compound **7e** was obtained starting from the 2,4-dihydroxybenzaldehyde **21**, following the general procedure. The crude was purified by trituration with hexane affording compound **7e** as a yellow solid. Yield 54%. M.p.: 100–102 °C. ^1^H-NMR (400 Hz; DMSO-*d*_6_) δ: 9.84 (s, 1H, -OH); 9.82 (s, 1H, -OH); 8.32 (s, 1H, -CH=N-); 7.28 (t, 2H, *J* = 8.0 Hz, Ar-H); 6.94 (dd, 1H, *J*_1_ = 8.0 Hz, *J*_2_ = 1.6 Hz, Ar-H,); 6.87–6.86 (m, 2H, Ar-H); 6.29–6.27 (m, 2H, Ar-H); 5.07 (s, 2H, -OCH_2_-); 3.75 (s, 3H, -OCH_3_). ^13^C-NMR (100 Hz; DMSO-*d*_6_) δ: 160.4, 158.8, 157.6, 147.2, 130.8, 129.3, 129.3, 113.6, 109.0, 107.6, 102.5, 75.0, 55.0.

(*E*)-3,5-Dihydroxybenzaldehyde *O*-(4-methoxybenzyl) oxime, **8a**.

Compound **8a** was obtained starting from the 3,5-dihydroxybenzaldehyde **17**, following the general procedure. The crude was purified by crystallization (CHCl_3_/hexane) affording compound **8a** as a yellow solid. Yield 60%. M.p.: 113–115 °C. ^1^H-NMR (400 Hz; DMSO-*d*_6_) δ: 9.41 (s, 2H, -OH); 8.05 (s, 1H, -CH=N-); 7.33 (dd, 2H, *J*_1_ = 6.7 Hz, *J*_2_ = 2 Hz, Ar-H); 6.92 (dd, 2H, *J*_1_ = 6.7 Hz, *J*_2_ = 2 Hz, Ar-H); 6.47–6.46 (d, 2H, *J* = 2.20 Hz, Ar-H); 6.24–6.23 (t, 1H, *J* = 2.20 Hz, Ar-H); 5.04 (s, 2H, -OCH_2_-); 3.75 (s, 3H, -OCH_3_). ^13^C-NMR (100 Hz; DMSO-*d*_6_) δ: 160.0, 158.6, 149.2, 133.5, 129.9, 129.5, 113.7, 105.0, 104.2, 75.0, 55.0.

(*E*)-3,4,5-Trihydroxybenzaldehyde *O*-(4-methoxybenzyl) oxime, **8b**.

Compound **8b** was obtained starting from the 2,3,4-trihydroxybenzaldehyde **18**, following the general procedure. The crude was purified by trituration with CHCl_3_, affording compound **8b** as a yellow solid. Yield 90%. M.p.: 123–125 °C. ^1^H-NMR (400 MHz; DMSO-*d*_6_) δ: 9.46 (s, 1H, -OH); 9.39 (s, 1H, -OH); 8.44 (s, 1H, -OH); 8.27 (s, 1H, -CH=N-); 7.33 (dd, 2H, *J*_1_ = 8.6 Hz, *J*_2_ = 2.8 Hz, Ar-H); 6.92 (dd, 2H, *J*_1_ = 8.6 Hz, *J*_2_ = 2.8 Hz, Ar-H); 6.76 (d, 1H, *J* = 8.50 Hz, Ar-H); 6.33 (d, 1H, *J* = 8.50 Hz, Ar-H); 5.02 (s, 2H, -OCH_2_-); 3.75 (s, 3H, -OCH_3_). ^13^C-NMR (100 Hz; DMSO-*d*_6_) δ: 159.0, 149.5, 148.3, 146.1, 132.7, 130.1, 129.4, 119.3, 113.7, 109.6, 107.7, 75.0, 55.1.

(*E*)-3,4-Dihydroxybenzaldehyde *O*-(4-methoxybenzyl) oxime, **8c**.

Compound **8c** was obtained starting from the 3,4-dihydroxybenzaldehyde **19**, following the general procedure. The crude was purified by trituration with hexane affording compound **8c** as a yellow solid. Yield 80%. M.p.: 85–87 °C ^1^H-NMR (400 Hz; DMSO-*d*_6_) δ: 9.31 (s, 1H, -OH); 9.17 (s, 1H, -OH); 8.03 (s, 1H, -CH=N-); 7.32 (d, 2H, *J* = 8.8 Hz, Ar-H); 7.03 (d, 1H, *J* = 1.96 Hz, Ar-H); 6.93 (d, 2H, *J* = 8.8 Hz; Ar-H); 6.83 (dd, 1H, *J*_1_ = 8.10 Hz; *J*_2_ = 1.96 Hz, Ar-H); 6.72 (d, 1H, *J* = 8.10 Hz, Ar-H); 5.01 (s, 2H, -OCH_2_-); 3.74 (s, 3H, -OCH_3_). ^13^C-NMR (100 Hz; DMSO-*d*_6_) δ: 159.0, 148.9, 147.6, 145.5, 129.9, 129.7, 123.2, 119.9, 115.5, 113.6, 112.8, 74.8, 55.0.

(*E*)-2,5-Dihydroxybenzaldehyde *O*-(4-methoxybenzyl) oxime, **8d**.

Compound **8d** was obtained starting from the 2,5-dihydroxybenzaldehyde **20**, following the general procedure. The crude was purified by trituration with hexane affording compound **8d** as a yellow solid. Yield 90%. M.p.: 93–95 °C. ^1^H-NMR (400 Hz; DMSO-*d*_6_) δ: 9.24 (s, 1H, -OH); 8.91 (s, 1H, -OH); 8.30 (s, 1H, -CH=N-); 7.33 (d, 2H, *J* = 8.4 Hz, Ar-H); 6.93 (d, 2H, *J* = 8.4 Hz, Ar-H); 6.90 (d, 1H, *J* = 2.8 Hz, Ar-H); 6.66 (dt, 2H, *J*_1_ = 12 Hz, *J*_2_ = 2.8 Hz, Ar-H); 5.04 (s, 2H, -OCH_2_-); 3.74 (s, 3H, -OCH_3_). ^13^C-NMR (100 Hz; DMSO-*d*_6_) δ: 159.0, 149.7, 148.9, 146.7, 130.0, 129.4, 118.7, 117.7, 117.0, 113.7, 112.2, 75.1, 55.1.

(*E*)-2,4-Dihydroxybenzaldehyde O-(4-methoxybenzyl) oxime, **8e**.

Compound **8e** was obtained starting from the 2,4-dihydroxybenzaldehyde **21**, following the general procedure. The crude was purified by trituration with hexane affording compound **8e** as a yellow solid. Yield 78%. M.p.: 71–73 °C. ^1^H-NMR (400 Hz; DMSO-*d*_6_) δ: 9.85 (s, 1H, -OH); 9.81 (s, 1H, -OH); 8.27 (s, 1H, -CH=N-); 7.32 (d, 2H, *J* = 8.8 Hz, Ar-H); 7.30–7.27 (m, 1H, Ar-H); 6.92 (d, 2H, *J* = 8.8 Hz, Ar-H); 6.30–6.27 (m, 2H, Ar-H); 5.01 (s, 2H, -OCH_2_-); 3.75 (s, 3H, -OCH_3_). ^13^C-NMR (100 Hz; DMSO-*d*_6_) δ: 160.4, 160.0, 157.8, 147.9, 130.0, 129.5, 129.2, 113.7, 109.0, 107.7, 102.4, 74.9, 55.0.

(*E*)-3,5-Dihydroxybenzaldehyde O-(2-chlorobenzyl) oxime, **9**.

Compound **9** was obtained starting from the 3,5-dihydroxybenzaldehyde **17**, following the general procedure. The crude was purified by trituration with hexane affording compound **9** as a yellow solid. Yield 65%. M.p.: 68–70 °C. ^1^H-NMR (400 Hz; CDCl_3_) δ: 8.05 (s, 1H, -CH=N-); 7.48–7.46 (m, 1H, Ar-H); 7.41–7.38 (m, 1H, Ar-H); 7.28–7.26 (m, 2H, Ar-H); 6.65 (d, 2H, *J* = 2.15 Hz, Ar-H); 6.38 (t, 1H, *J* = 2.15, Ar-H); 5.32 (s, 1H, -OCH_2_-). ^13^C-NMR (100 Hz; DMSO-*d*_6_) δ: 158.6, 149.9, 135.1, 135.2, 132.6, 130.5, 129.7, 129.3, 127.2, 105.1, 104.4, 72.5.

(*E*)-3,5-Dihydroxybenzaldehyde O-(3-chlorobenzyl) oxime, **10**.

Compound **10** was obtained starting from the 3,5-dihydroxybenzaldehyde **17**, following the general procedure. The crude was purified by trituration with hexane affording compound **10** as a yellow solid. Yield 54%. M.p.: 116–118 °C. ^1^H-NMR (400 Hz; DMSO-*d*_6_) δ: 9.42 (s, 2H, -OH); 8.13 (s, 1H, -CH=N-); 7.44–7.34 (m, 4H, Ar-H); 6.46 (d, 2H, *J* = 2.20 Hz, Ar-H); 6.24 (t, 1H, *J* = 2.20 Hz, Ar-H); 5.14 (s, 2H, -OCH_2_-). ^13^C-NMR (100 Hz; DMSO-*d*_6_) δ: 158.6, 149.9, 140.4, 133.2, 132.9, 130.2, 127.6, 127.6, 126.5, 105.0, 104.4, 74.2.

(*E*)-3,5-Dihydroxybenzaldehyde O-(4-chlorobenzyl) oxime, **11**.

Compound **11** was obtained starting from the 3,5-dihydroxybenzaldehyde **17**, following the general procedure. The crude was purified by trituration with hexane affording compound **11** as a yellow solid. Yield 55%. M.p.: 125–127 °C. ^1^H-NMR (400 Hz; DMSO-*d*_6_) δ: 9.42 (s, 2H, -OH); 8.10 (s, 1H, -CH=N-); 7.45–7.43 (m, 2H, Ar-H); 7.42–7.40 (m, 2H, Ar-H); 6.45 (d, 2H, *J* = 2.20 Hz, Ar-H); 6.23 (t, 1H, *J* = 2.20 Hz, Ar-H); 5.12 (s, 2H, -OCH_2_-). ^13^C-NMR (100 Hz; DMSO-*d*_6_) δ: 158.6, 149.7, 136.8, 133.3, 132.3, 129.9, 128.3, 105.0, 104.4, 74.3.

### 2.2. Functional Studies

#### 2.2.1. Aldose Reductase Inhibitory Activity

##### Recombinant Expression of Aldose Reductase in *E. coli*

Design of the expression plasmid.

The plasmid expression carries a T7 promoter/terminator, a *hexa*-histidine (6His) tag for nickel affinity purification, a Tobacco Etch Virus (TEV) protease cleavage site (ENLYFQ/G) and a gene encoding the ALR2. The 6His tag is placed between the fusion partner (DsbC) and the TEV site [20]. The synthetic gene optimized for recombinant expression of ALR2 in *E. coli* (BL21 5DE3 pLysS strain) was ordered from Eurofins.

Expression of aldose reductase.

The expression strain BL21 (DE3) pLysS was obtained after a heat-shock transformation of competent cells with the expression plasmid. Transformed cells were used to inoculate pre-cultures containing 15 mL of LB media supplemented with ampicillin overnight at 37 °C. The following morning, a Fernbach flask containing 1l of ZYP-5052 automedium (25 mM Na_2_HPO_4_, 25 mM KH_2_PO_4_, 50 mM NH_4_Cl, 5 mM Na_2_SO_4_, pH 6.7, 5% glycerol, 0.5 g glucose, 2 g α-lactose, 10 g N-Z-amine AS, 5 g yeast extract, and 1 mM MgSO_4_) and 100 µg/mL ampicillin was inoculated with the overnight pre-culture of BL21 (DE3) pLysS at 0.05 O.D. at 600 nm. ZYP-5052 medium is a buffered complex medium containing glucose, lactose, and glycerol formulated to induce protein expression after glucose depletion [21]. Expression was performed using a standardized two-step process. In the first part of fermentation, cells were grown at 37 °C to quickly reach the glucose depletion phase just before the induction. After that step at 0.9 O.D. at 600 nm (4 h), the temperature was lowered to 20 °C for 18 h to favor soluble protein expression. After 18 h, cells were pelleted by centrifugation and re-suspended in 80 mL of lysis buffer containing 100 mM Tris HCl pH 8, 150 mM NaCl, and 5% glycerol. 2 μL of benzonase and 0.1 M MgCl_2_ were added to the suspension. After 30 min at 4 °C with gentle stirring, the cells were lysed by two passes at 1.5 kbar on EMULSIFLEX. After centrifugation for 45 min at 18.000 rpm at 4 °C, the soluble extract contained in the supernatant was separated from the insoluble fraction contained in the pellet.

Purification and TEV cleavage of DSBC-aldose reductase.

The soluble extract was filtered through 0.22 μM and loaded on a 5 mL HisTrap™FF column (GE Healthcare) with a 1 mL/min flow rate. Buffers used for purification were a binding buffer containing 100 mM tris-HCl pH 8, 150 mM NaCl, 5 mM Imidazole, 5% glycerol, and elution buffer containing 100 mM Tris-HCl pH 8, 150 mM NaCl, 500 mM imidazole, and 5% glycerol. The 6His-tagged fusion protein was eluted with a linear gradient (0 to 100% B in 30 min at a flow rate of 1 mL/min. The fractions containing the 6His-tagged fusion protein were pooled and dialyzed for 3 h against a buffer containing 100 mM Tris HCl pH 8, 150 mM NaCl using a Spectra/Por^®^ Dialysis Membrane (MWCO: 3500). The protein of interest was then cleaved with 10% (*w*/*w*) TEV protease overnight at 4 °C.

Purification of aldose reductase.

The TEV cleavage was loaded on a 5 mL HisTrap^TM^FF column (GE Healthcare) with a 1 mL/min flow rate. The protein DSBC and TEV protease with 6His-tag were bound on the column. The ALR2 was recovered in the unrestrained fraction and dialyzed for 3 h against a buffer containing 100 mM Tris HCl pH 8, 150 mM NaCl using a Spectra/Por^®^ Dialysis Membrane (MWCO: 3500). The last step was a purification of ALR2 on size-exclusion chromatography (*SEC*) Sephacryl ^®^ S-100 HR GE Healthcare in buffer 100 mM Tris HCl pH 8, 150 mM NaCl (Figures S1 and S2, Supplementary Materials). ALR2 was concentrated to 1.47 mg/mL (41 µM).

Inhibition of aldose eductase.

The human recombinant ALR2 enzyme activity was determined spectrophotometrically by monitoring the change in absorbance at 340 nm, due to the oxidation of the NADPH cofactor catalyzed by the enzyme. According to a previously reported protocol, the enzymatic assay was performed in a 96-multiwell plate (250 μL) [7]. The reaction mixture, containing sodium phosphate buffer pH = 6.2 (0.1 M), NADPH (0.15 mM), ALR2 (0.050 μM), and D,L-glyceraldehyde (10 mM), was monitored for 5 min at 30 °C using a Victor3 Perkin Elmer Multilabel Plate Reader. The variation of pyridine coenzyme concentration versus time was analyzed through the WorkOut program. All the new synthesized compounds were solubilized in DMSO (10 mM) and screened at a final concentration of 100 μM. Assays were made in triplicate, and Tolrestat was used as the reference drug. Compounds **6b**, **7b**, and **8b**, showing the best inhibitory activity at 100 μM, were further tested at additional concentrations within 50 μM and 10 μM.

#### 2.2.2. Anti-Oxidant Activity

##### Thiobarbituric Acid Reactive Substances (TBARS) Assay

A TBARS assay was carried out according to a previously reported protocol [13], using freshly isolated rat brain. Stock solutions of 10 mM test compounds were obtained in DMSO, then diluted to test samples with phosphate buffer pH 7.4. In a falcon tube, 100 µL of FeCl_3_ (20 µM) and 100 µL of ascorbic acid (100 µM) were added to 100 µL of rat brain homogenate, in the absence or presence of 100 µL of test compounds. The resulting solution was diluted to 1 mL with phosphate buffer pH 7.4 and incubated at 37 °C for 30 min under slight stirring. Then, 0.5 mL of thiobarbituric acid (1% *w*/*v* in 0.05 N NaOH) and 0.5 mL of 25% *v*/*v* HCl were added, and the mixture was boiled for 10 min. After being cooled in an ice-cold water bath, the mixture was added with 3 mL of n-butanol and centrifuged at 2000× *g* for 10 min. The absorbance of the organic phase was recorded at 532 nm using a SPECTROstarNano (200–1000 nm) UV/Vis spectrophotometer. The synthesized compounds were tested in triplicate at 100 μM, 10 μM, and 1μM concentrations. Resveratrol and its dimethoxy-derivative pterostilbene (Sigma–Aldrich) were used as the reference compound. TBARS was expressed as nmoles of malondialdehyde (MDA)/10 mg of rat brain tissue (wet weight), using a reference curve obtained with 1,1,3,3-tetramethoxypropane as the standard [22].

##### DPPH Assay

The DPPH assay was carried out in a 96-microwell plate (200 μL), following a previously described protocol [23]. Briefly, 195 μL of freshly prepared DPPH solution (60 μM in MeOH) was added with 5 μL of either test compounds or MeOH as the control. The plate was incubated in the dark for 1 h, at r.t. The absorbance was measured at 517 nm using a SPECTROstarNano (200–1000 nm) UV/Vis spectrophotometer. The synthesized compounds were tested in triplicate at 100 μM and 10 μM. Resveratrol (Sigma–Aldrich) was used as the reference standard. The percentage of the antioxidant activity (AA) of the compounds was determined according to the following Equation (1):

%AA = ((Abs_DPPH_ − Abs_Blank1_) − (Abs_sample_ − Abs_Blank2_)/Abs_DPPH_ − Abs_Blank1_) × 100 (1)

where Abs_DPPH_ is the absorbance of DPPH solution, Abs_Blank1_ is the absorbance of MeOH, Abs_sample_ is the absorbance of DPPH solution containing the test compound, and Abs_Blank2_ is the absorbance of test compound solution without DPPH.

### 2.3. Computational Investigations

#### 2.3.1. Docking Studies

##### System Preparation

The X-ray crystallized ALR2 structures, including 2FZB [24], 4JIR [25], 4IGS [14], and 4JII [25] conformations, were obtained from the RCSB Protein Data Bank. Each protein conformation was prepared by removing the co-crystallized molecules, including Tolrestat, Epalrestat, JF0064, and Zopolrestat, water, and sodium, except nicotinamide adenine dinucleotide phosphate (NADP^+^). The UCSF ChimeraTool-1.13.114 [26,27] was used to obtain four different ALR2 conformations and the test compounds **6b**, **7b**, and **8b** for molecular docking calculations.

##### Molecular Docking Calculations

Docking studies were performed on the prospective inhibitors, including **6b**, **7b**, and **8b** (Table 1). The ligand compounds were drawn in MarvinSketch application-21.17 [28,29] and converted to mol2 format. Each compound was manually assessed in the Molegro Molecular View-7.0.0 (MMV) [30] to ascertain correct bond angles and hybridization state. Ligands were minimized using the steepest descent method and GAFF force field in Avogadro-1.2.0n [31,32]. The compounds were individually docked into the active site of the 2FZB, 4JIR, 4IGS, and 4JII aldose reductase protein. However, this study reported the grid box parameters for the systems involving 4JII because the conformation gave better binding energies with the inhibitors of interest. These were specified using AutodockTool-1.5.6 software [33,34] and defined the center as X = −6.304, Y = 6.647, and Z = 19.394 with dimensions X = 58, Y = 46, and Y = 66. The grid box was centered around TRP112 native conformation as it corresponds to the residual trio (TYR49, HIS111, and TRP112) of the anion pocket and specificity binding site of human ALR2 protein [24]. The structural and visual analyses of the 4JII ALR2-6b, ALR2-7b, and ALR2-8b were carried out using BIOVIA Discover studio Visualizer Software-4.0 [35,36].

#### 2.3.2. Molecular Dynamics Studies

##### Retrieval and Preparation of Aldose Reductase

The X-ray crystallized ALR2 monomeric structure 4JII [25] was obtained from the RCSB Protein Data Bank. Compounds **6b** (working code FB7), **7b** (working code FB8), and 8b (working code FB6) were drawn in the MarvinSketch tool, and their geometries were optimized with the General Amber Force Field (GAFF) in Avogadro tool-1.2. On [31]. Both protein and inhibitors were prepared using the Chimeratool-1.14 [26] and the AutoDockTool-1.5.6 [37]. Moreover, the systems were opened and saved on the Molegro Molecular Viewer-2.5 [38] for the molecular dynamics simulation study. The preparation of the unbound and ligand-bound complexes involves removing nonstandard molecules, including water, NAP+, and sodium [27]. Figure S3 (Supplementary Materials) provides the structures of the ALR2 conformation showing some active site residues (A) and the chemical structures of the test inhibitors.

##### Molecular Dynamics (MD) Simulations

The MD simulation of unbound and bound ALR2 systems were carried out with Amber code using the graphic processor unit (GPU)-Compute Unified Device Architecture (CUDA) accelerated version of the AMBER18 package at the Lengau CHPC server [39] with integrated PMEND engine and LEAP modules [40]. The FF18SB force field variant was applied to describe the inhibitor–ALR2 enzyme complexes. The antechamber was used to add the atomic partial charges for the ligands using the restrained electrostatic potential (RESP) and the General Amber Force Field (GAFF) procedures [41]. The Leap module of Amber 18 enabled the addition of hydrogen atoms, sodium (Na^+^), or chloride (Cl^−^) counter ions to the systems for neutralization [42]. The complexes, implicitly, were suspended within an orthorhombic box of TIP3P water molecules to contain all the atoms within 8 Å of any box edges.

The systems were initially minimized for 2500 steps with 500 kcal/mol Å^2^ restraint potential, and a whole minimization step of 5000 steps was further run without restraint using the conjugate algorithm. The heating step was carried out in a canonical ensemble condition (NVT) by a gradual heating simulation (0 to 300 K) executed for 5 ps while several atoms and volume were fixed. Moreover, the systems-containing solutes were imposed with a potential harmonic restraint of 10 kcal/mol Å^2^ and a collision frequency of 1.0 ps^−1^. An equilibration estimation of 1 ns was carried out while keeping the operating temperature (300 K) constant. Moreover, the number of atoms and pressure were constant, depicting an isobaric–isothermal ensemble (NPT). Using the Berendsen barostat, the complex pressure was kept at 1 bar [43,44]. The SHAKE algorithm was employed to construct the hydrogen atom bonds [43]. Coordinates and trajectories were printed and analyzed every 1 ps using the PTRAJ module available in AMBER18GPU [42,45]. The stabilities and flexibilities of the free protein and ligand-bound complexes were investigated statistically by estimating the averages RMSD [39] and RMSF [34], respectively. Furthermore, principal component analysis (PCA) [46] was calculated to unravel the extent of the enzyme’s atomic displacement. In addition, the dynamic cross-correlation matrix data were computed to gain insights into the strength and similarities of the linear relationship between the inter-residues of the bound and unbound systems. The detailed methods for obtaining RMSD, RMSF, PCA, and SASA [39,47] have been frequently reported in the literature, including our previous research works.

##### Binding Free Energy Computations

Over the years, molecular dynamics have been widely applied to study the mechanism of several biological processes by computing differences in the binding free energy [48]. The binding affinities of the bound and unbound systems were obtained by computing the binding free energies using the molecular mechanics/GB surface area method (MMGBSA) [49]. The detailed protocols of the methodology can be found in our previous works [39]. The free energies were calculated based on the average of over 40,000 snapshots extracted from the simulation trajectories. The estimated free binding energy, ΔG, for each molecular system, including the complex, inhibitor, and protein, can be given as:

ΔG_bind_ = G_complex_ − G_protein_ − G_inhibitor_ (2)

ΔG_bind_ = E_gas_ + G_sol_ − TS (3)

E_gas_ = E_int_ + E_vdw_ + E_ele_ (4)

G_sol_ = G_GB_ + G_SA_ (5)

G_SA_ = γSASA (6)

ΔG_bind_ is the summation of the gas phase, while E_gas_ denotes the gas-phase energy. The solvation energy (G_sol_) < the entropy (TΔS). E_int_ term = the internal energy, E_vdw_ term = the van der Waals energies, and E_ele_ = the electrostatic interaction energies. The E_gas_ was obtained from the AMBER18 FF14SB force fields module. The energy of the polar and the non-polar states was used to account for the solvation free energy. The product of the solvent accessible surface area (SASA) and a water probe radius of 1.4 Å was obtained to compute the non-polar solvation energy (G_SA_). The S term is the total entropy of the system, which is often calculated using the normal mode method incorporated in the AMBER18 module [50].

## 3. Results and Discussion

**Scheme 1:**
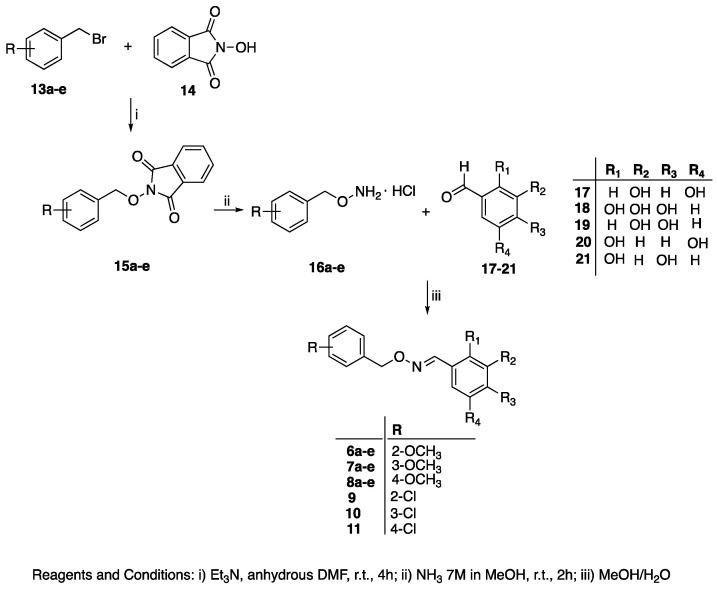
Synthesis of the (*E*)-benzaldehyde *O*-benzyl oximes **6a–e**, **7a–e**, **8a–e**, **9–11**.

The synthesis of the target (*E*)-benzaldehyde *O*-(benzyl)-oximes **6a–e**, **7a–e**, **8a–e**, and **9–11** (Table 1) was carried out as outlined in Scheme 1.

The O-(arylmethyl)hydroxylamine hydrochlorides **16a–e** were synthetized as previously described by a Mitsunobu reaction between the suitably substituted benzyl bromide **13a–e** and the N-hydroxyphthalimide **14**, followed by the removal of the protecting phthalimido group from the key intermediates **15a–e**, carried out with ammonia solution 7N in MeOH [17,51,52]. Treatment of **16a–e** with the opportune aldehydes **17–21**, in methanolic solution and at room temperature, afforded the desired (*E*)-benzaldehyde *O*-benzyl oxime derivatives **6a–e**, **7a–e**, **8a–e,** and **9–11** as the only geometric isomers. The correct configuration of the products was attributed by their ^1^HNMR spectra on the basis of the chemical shift value of the diagnostic iminic proton, ranging from 8.46 to 8.03 ppm as reported by the literature for the *E* form [52]. The IR spectra of compounds **6a–e**, **7a–e**, **8a–e,** and **9–11** were recorded as well. The obtained spectral signatures are listed in Table S1 (Supplementary Materials). All the synthesized derivatives were investigated in vitro for their dual activity. As for the inhibitory efficacy against the target ALR2, compounds were tested at 100 μM using a human recombinant enzyme. Results obtained, summarized in Table 2, highlight the prominent role of the polyhydroxy decoration of the A ring over the substituents on the B ring in determining the activity of the compounds. In fact, the 3,5-dihydroxy substitution pattern was detrimental, regardless of the residue on the B phenyl ring. See for example derivatives **6a**, **7a**, **8a**, and **11**, which were devoid of any activity. The 2,4-, 2,5-, and 3,4-dihydroxy functionalization conferred some inhibitory efficacy, which was shown to be of the same order regardless of hydroxy groups’ position on the A ring and the substituent of B ring. On the contrary, the 2,3,4-trihydroxy functionalization proved to be the most promising. Indeed, compound **6b**, carrying a 2-methoxybenzyl moiety, almost halved ALR2 activity, and an even better efficacy was shown by **7b** and **8b**, bearing 3-methoxy and 4-methoxy residues on the B phenyl ring, respectively. They both stood out as the best performing compounds in the whole series, thus also emphasizing the importance of the substituent on the B ring to achieve the best interaction with the catalytic binding site of the enzyme.

Regarding the anti-oxidant properties, compounds were tested in both TBARS and DPPH assays.

**Figure 2:**
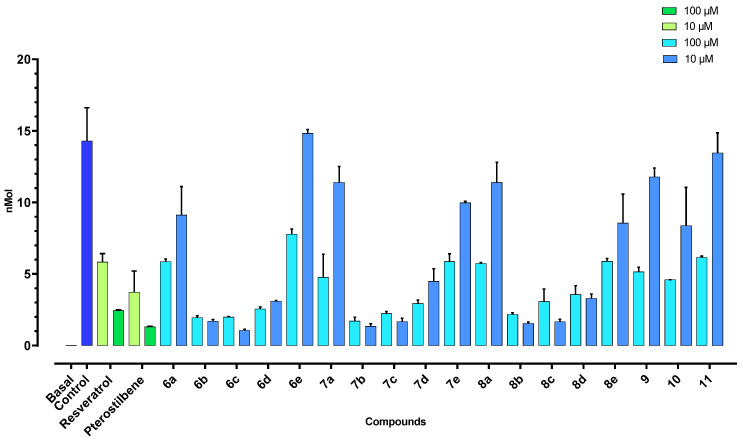
Effects of (*E*)-benzaldehyde *O*-benzyl oximes **6a–e**, **7a–e**, **8a–e**, and **9–11**, resveratrol, and pterostilbene on the production of thiobarbituric acid reactive substances (TBARS) in rat brain homogenate.

The TBARS assay highlights the ability of the compounds to block the hydroxy radical-dependent lipid peroxidation induced by the oxidant system Fe(III)/ascorbic acid in rat brain homogenate, and results in malondialdehyde (MDA) as the ultimate metabolite. Tested at 100 μM, all the synthesized compounds showed a relevant anti-oxidant efficacy (Figure 2). Moreover, in this case, the reciprocal arrangement of the hydroxy substituents on the A phenyl ring of the derivatives proved to be a crucial determinant, remarkably affecting their functional potency. In particular, the 2,3,4-trihydroxy, 3,4-dihydroxy, and 2,5-dihydroxy combinations turned out to perform the best. Indeed, for **6b–d**, **7b–d**, and **8b–d**, the obtained amounts of MDA (nmol *per* mg) in brain tissue were comparable or even lower than those attained with both resveratrol and its methoxylated derivatives, pterostilbene, used as the reference compounds (Figure 2). Significantly, **6b–d**, **7b,c**, and **8b–d** showed an excellent activity when tested at 10 μM (Figure 2), with **7b** still active even at 1 μM (Figure S4, Supplementary Materials). On the contrary, the anti-oxidant potency of the natural compounds came down on a dose-dependent basis.

**Figure 3:**
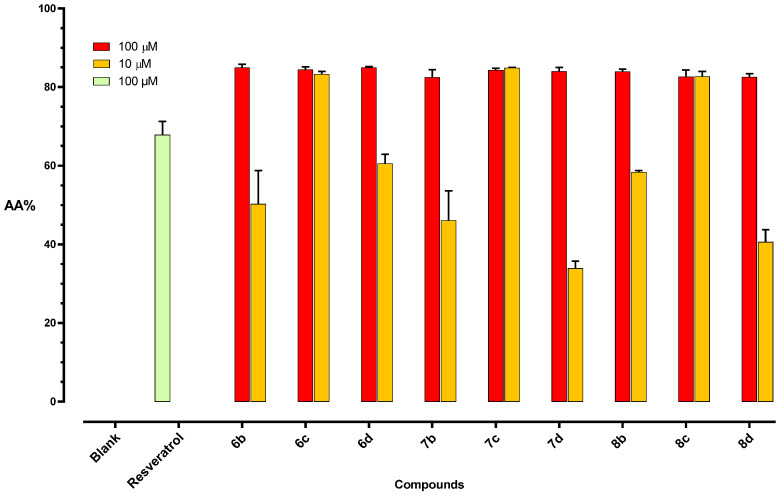
Radical scavenging capacity of (*E*)-benzaldehyde *O*-benzyl oximes **6b–d 7b–d**, **8b–d**, and resveratrol, expressed as a percentage of anti-oxidant activity (AA%).

In light of the relevant results from the TBARS assay, compounds **6b–d**, **7b–d**, and **8b–d** were also investigated for their free radical scavenging capacities by the DPPH assay. The selected derivatives showed remarkable efficacy (Figure 3). Notably, when tested at 100 µM concentration, they proved to be more potent than the reference resveratrol. As for derivatives **6c**, **7c**, and **8c**, the same efficacy was maintained even at 10 µM test concentration, demonstrating that, among the explored polyhydroxy combinations, the 3,4-dihydroxy catechol arrangement on the A ring ensures the achievement of the best free-radical quenching properties.

The promising ALR2 inhibitory activity of **6b**, **7b**, and **8b** was rationalized through molecular docking studies, thereby representing an increasingly valuable tool in drug design [53]. The catalytic binding site of ALR2 is characterized by a high degree of plasticity [54,55,56]. From a functional point of view, such a feature provides a broad substrate specificity to the enzyme. However, in structural terms, it implies the existence of different crystal arrangements obtained in the presence of different compounds, ALR2 being able to modify the conformation of its binding site by an induced-fit adaptability to the ligands. Therefore, to accomplish a reliable docking investigation, different conformations should be considered.

To investigate the SARs of the novel synthesized compounds, four different enzyme conformations were examined, identified by the PDB codes 2FZB [24], 4JIR [25], 4IGS [14], and 4JII [25], and obtained by different authors co-crystallizing the known inhibitors Tolrestat, Epalrestat, JF0064, and Zopolrestat, respectively.

As reported in Table 3, docking calculations showed that 4JII conformation allowed to achieve the highest binding energy, indicating that ligands are more embedded in this active pocket than the other ALR2 reported structures. Moreover, the highest binding affinities were obtained for the ALR2-**7b** complex across all the investigated enzyme–ligand complexes. The resulting energies, including −7.61 kcal/mol, −8.1 kcal/mol, −8.4 kcal/mol, and −9.4 kcal/mol, demonstrate the highest affinity of the compound compared to the parents **6b** and **8b**, regardless of conformation used.

Starting from the evidence, we focused on the 4JII ALR2 structure to investigate the binding forces and interaction types of the protein–inhibitor systems, to gain further insights into the binding and inhibitory mechanisms of the prospective inhibitors **6b**, **7b**, and **8b**.

Figure S5 (Supplementary Materials) highlights the ALR2 pocket residues interacting with the pharmacophoric portions of inhibitor **6b**, including TRP21, VAL48, TRP112, VAL301, LEU302, and PHE321. The observed interactions were highly hydrophobic, indicating the extent to which the ligand was buried within the enzyme active site.

Similar results were also achieved by docking compound **8b** into the same enzyme pocket (Figure S6, Supplementary Materials). Besides the already mentioned VAL48, TRP112, VAL301, and LEU302, ASP300 and PHE123 were recruited to obtain the complex. Compound **8b** was kept within the ALR2 binding site by highly hydrophobic interactions. Still, additional hydrogen bonds could also be formed between the 3-hydroxy group on the A ring of the compound and both VAL301 and LEU302, which provides the reason for the better inhibitory efficacy of **8b** compared to **6b**.

As for derivative **7b** (Figure S7, Supplementary Materials), the ALR2 residues contacting the inhibitor included TRP21, VAL48, TYR49, HIS111, TRP112, CYS299, VAL301, and LEU302. Similar to the parents **6b** and **8b**, the observed interactions were mainly hydrophobic. However, in addition to those previously described, classical hydrogen bond interactions involving VAL301 and a sulphur bond engaging CYS299 were also observed, which allowed **7b** to be placed into the enzyme binding site. Moreover, differently from **6b** and **8b**, compound **7b** can also interact with the NAD^+^ co-factor, due to the 3-methoxy group on the B phenyl ring. On the whole, the occurring interactions maximize the binding of **7b** within the target enzyme, rationalizing the observed inhibitory efficacy and justifying the highest binding energy resulting from the ALR2-**7b** complex.

**Figure 4:**
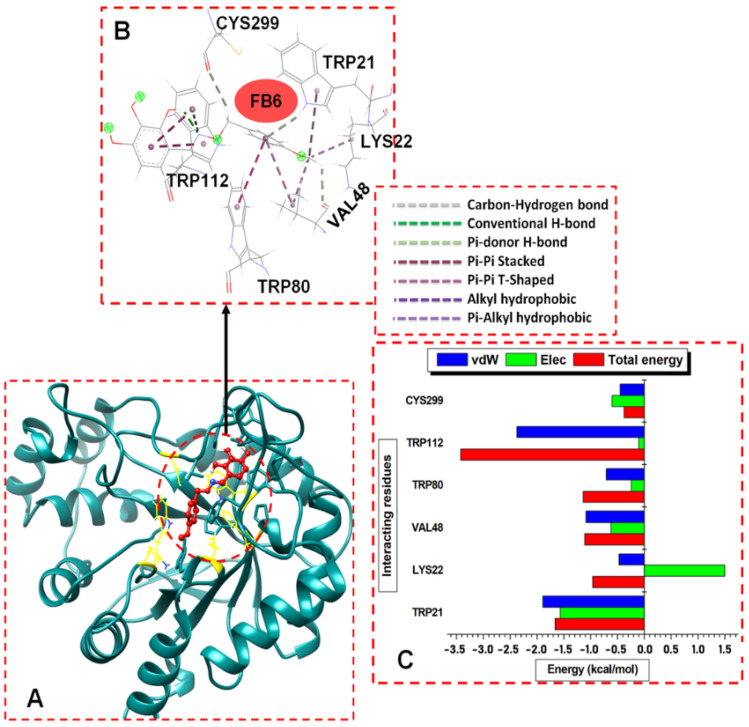
(**A**) Structural view of **8b** (working code FB6) bound to ALR2 enzyme active site. (**B**) Visual illustration of the ALR2-**8b** interaction network (color online). (**C**) Per-residue energy contributions to the ALR2-**8b** complex.

**Figure 5:**
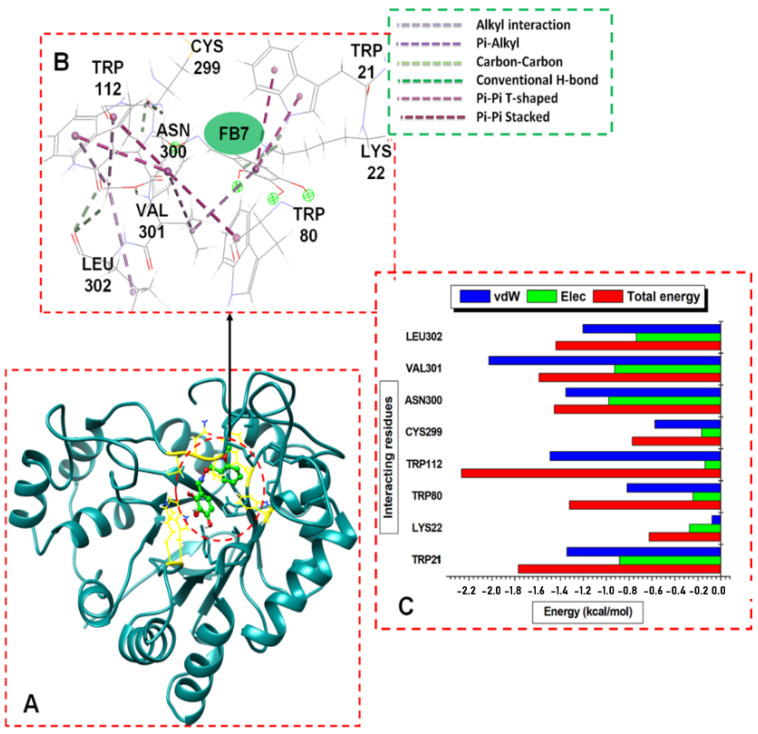
(**A**) Structural view of **6b** (working code FB7) bound to ALR2 enzyme active site. (**B**) Visual illustration of the ALR2-**6b** interaction network (color online). (**C**) Per-residue energy contributions to the ALR2-**6b** complex.

**Figure 6:**
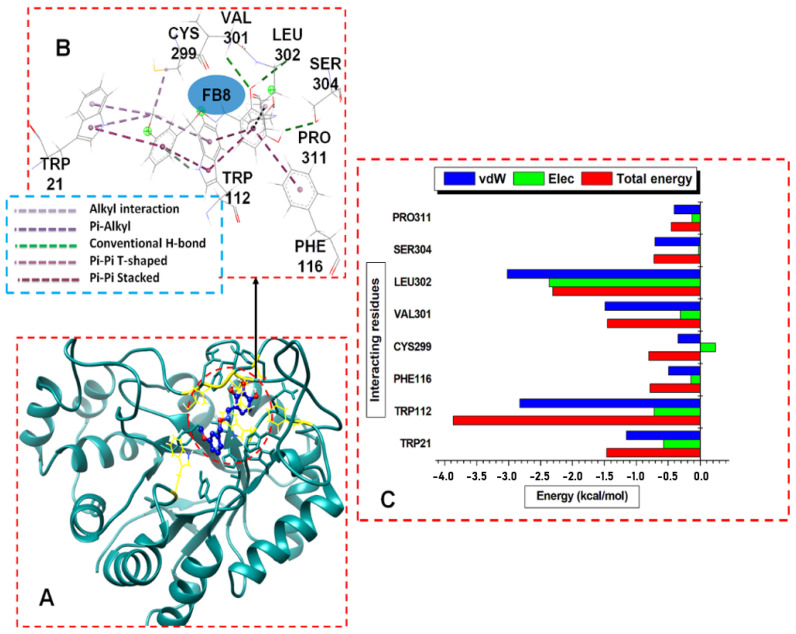
(**A**) Structural view of **7b** (working code FB8) bound to ALR2 enzyme active site. (**B**) Visual illustration of the ALR2-**7b** interaction network (color online). (**C**) Per-residue energy contributions to the ALR2-**7b** complex.

The obtained ALR2–inhibitor complexes were compared with the unbound ALR2 system by evaluating the motion and dynamic of their structural behaviors using the all-atom molecular dynamics (MD) simulation for 200 ns. The per-residues energy decomposition (PRED) analysis identified the interacting residues and their contributing energies to the binding affinity [57]. The contributions of the ALR2 residues were unraveled on the Discovery Studio Viewer, which individually allowed insights into the interacting network of residue-inhibitor complexes ALR2-**6b**, ALR2-**7b**, and ALR2-**8b** (Figure 4A, Figure 5A and Figure 6A). The PRED of ALR2 residues revealed some interactions with the ligands at the active site during the 200 ns MD simulations. Figure 4B, Figure 5B and Figure 6B show that the inhibitors adopted favorable orientations, whereby the functional groups such as phenyl (-C_6_H_5_), methoxy (-OCH_3_), and hydroxyl (-OH) interacted with the residues by various covalent hydrogen bonds (C–H, pi-donor H-bond, H–H) and hydrophobic interactions, including pi–pi stacked, pi–pi T-shaped, alkyl, and pi–alkyl bonds. In particular, in the case of **8b**, the methoxy group of the ligand formed strong alkyl and pi–alkyl interactions with the backbones of LYS22 and VAL48 and, to expatiate, the pi-donor H-bond with VAL48. The CYS299 oxygen interacted with the CH_2_-O group of the ligand, to form a C-H bond, while TRP80 and TRP112 formed pi–pi T-shaped and pi–pi stacked, with phenyl of trihydroxybenzaldehyde and methoxybenzyl groups of **8b**, respectively.

The notable differences in the interactions across the three inhibitors are the type and the quality of hydrogen bonds formed within each complex. For instance, ALR2-**8b** and ALR2-**7b** showed more H-bonds and hydrophobic interactions than ALR2-**6b**, due to the higher residue numbers involved in the interaction. We also observed that **7b** formed three strong non-polar covalent H-bonds with VAL301, LEU302, and SER304, which may have contributed to the high binding energy of the ALR2-**7b** complex (Figure 4B). Although the inference may not be accurate for all protein–ligand interactions, the binding free energy calculated for each complex provided in Table 1 justified this assertion. The resonating phenyl rings of the trihydroxybenzaldehyde and methoxybenzyl moieties formed pi–alkyl bonds, pi–pi stacked, and T-shaped bonds. Other strong interactions were the different polar and non-polar hydrogen bonds formed, especially with compound **7b**.

Furthermore, we obtained residue-based contributions from the decomposition of total binding energy (BPE) using the MM/GB(PB) method. The van der Waals (vdW) and electrostatic (Elec.) interactions were computed. The two forces showed that the residues and energy contributed more to the total binding energy. The stability of a protein–ligand system depends on intermolecular forces between the interacting residues and the ligands and, subsequently, the binding free energy (delta G) [58]. Figure 4C, Figure 5C and Figure 6C showed the individual hydrophobic residue pocketing **8b**, **6b**, and **7b**, respectively, revealing their energy contributions. The **8b** interacted with TRP21, LYS22, VAL48, TRP80, TRP112, and CYS299. Similarly, **6b** and **7b** interacted with TRP21, TRP112, and CYS299. Other residues that interacted with **6b** included LYS22, TRP80, ASN300, VAL301, and LEU302, while **7b** interacted with more residues, including PHE116, SER304, and PRO311.

The residues found in TRP21 and TRP112 and the energy contributions to the binding of the ligands were significant, as seen in Figure 4C, Figure 5C and Figure 6C. In contrasting these MD results with the molecular docking results, the findings showed that **8b**- and **7b**-bound ALR2 complexes had higher average energies than the **6b**-bound complexes; the contributed residues from TRP21 and TRP112 had higher binding energies to than **6b**.

The residues and their respective energy (kcal/mol) in parentheses, TRP112 (−3.3), TRP21 (−1.7), TRP80 (−1.0), VAL48 (1.0), and LYS22 (0.8), contributed most of the interacting energies to the **8b** binding, while CYS299 (−0.4) contributed less energy. Similarly, TRP112 (−3.8), LEU302 (−2.4), VAL301 (−1.5), and TRP21 (−1.45) contributed higher energies to **7b** binding, while CYS299 (−0.8), PHE116 (−0.7), SER304 (−0.6), and PRO311 (−0.5) had less energy contributions. The residues that contributed higher energies to the binding of **6b** included TRP112 (−2.4 kcal/mol), TRP80 (−1.0), TRP21 (−1.8), VAL301 (−1.9), ASN300 (−1.6), LEU302 (−2.4), and TRP80, while CYS299 (−0.8) and LYS22 (−0.6) contributed less energy. Moreover, the van der Waals interactions were high compared to the electrostatic interactions across the complexes, particularly for the system docked with compounds **8b** and **7b**.

The computational MM/GB(PB)SA approach [59] was used to obtain the total binding free energy (BFE) of the complexes (ALR2-**6b**, ALR2-**7b**, and ALR2-**8b**). The BFE analysis estimates distinct energy contributions within the protein binding pocket and orientation with the best intermolecular interactions. The approach provides insights into the binding affinities of the compounds. Table 4 showed the total binding free energy (kcal/mol) values calculated for the ALR2-**6b** (−25.40), ALR2-**7b** (−34.51), and ALR2-**8b** (−31.48) complexes. The binding energies for **6b**, **7b**, and **8b** calculated from the molecular docking comparatively disagreed with the experimental data, unlike the binding energies calculated from the molecular dynamics studies. Simply put, the inhibition of the ALR2 enzyme by **8b** (67% inhibition) and **7b** (63% inhibition) are more potent and promising than **6b** (44% inhibition). The molecular docking results revealed that binding energies of **8b**, **6b**, and **7b** increase in ascending order, respectively. The MD studies showed that **8b** and **7b** have higher binding energies. Likewise, the PRED analysis and the ALR2-ligand interaction networks expatiated these findings, as seen in the immediate section.

The molecular dynamics simulations method is more suitable to achieve a stable protein–ligand complex than molecular docking, while combining the two methods is more reliable [34,60]. Hence, we inferred that the binding affinity of the ligands, especially **8b,** was not stable during molecular docking studies since the binding energy of the ALR2-**8b** was greater than **6b** in the MD findings. Therefore, the results showed that the systems’ electrostatic energies (ΔEelec) were lower than the van der Waal (ΔE_vdW_) forces across the three complexes.

**Figure 7:**
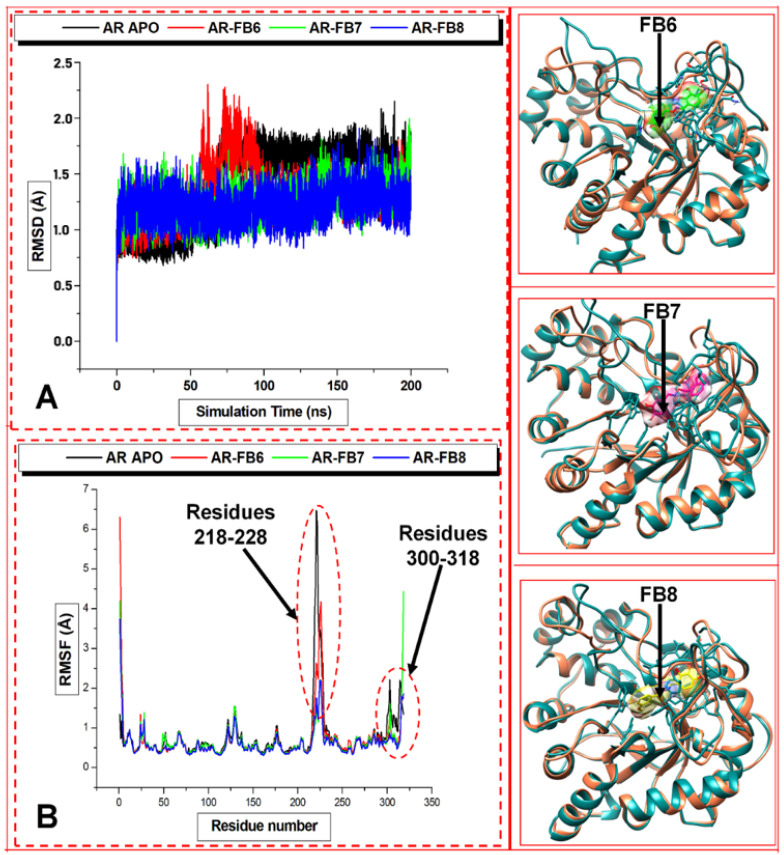
RMSD (**A**) and RMSF (**B**) plots for the unbound protein (black, working code AR–Apo), and the complex with **8b** (red, working code AR-FB6), **6b** (green, working code AR-FB7) and **7b** (blue, working code AR-FB8). The structures C, D, and E are simulated ALR2-**8b**, ALR2-**6b**, ALR2-**7b** complexes at 200 ns superimposed with the ALR2–Apo system.

The molecular dynamics (MD) simulation method was carried out to determine the atomic and molecular movements over 200 ns. Root mean square deviation [61] (RMSD) was used to estimate the differences in the C-α backbones of the Apo (initial) structural conformation and the bound ALR2 complexes. The observed deviations quantified the conformational stability of the protein during the MD simulations. Recall, more minor deviations indicate a more stable protein or protein–complex structure. The C-α backbone RMSD was determined for 200 ns simulation time to evaluate the stability of the unbound and bound ALR2 systems. Figure 7A shows that the four systems equilibrated immediately after simulation. Still, ALR2–Apo and ALR2-**8b** visibly fluctuated at about 60 ns with a small RMS deviation increment and remained stable again from 90 ns. In contrast, ALR2-**6b** and ALR2-**7b** appeared to maintain overall stability almost throughout the 200 ns. However, the average RMSD values for ALR2–Apo (1.41 Å), ALR2-**8b** (1.3 Å), ALR2-**6b** (1.25 Å), and ALR2-**7b** (1.21 Å) showed deviations in descending order, although this was minimal.

Furthermore, root mean square fluctuations (RMSF) of the unbound and bound ALR2 systems were computed. RMSF analysis provides insights into the protein’s flexibility [61]. Similarly, the RMSF indicated higher flexibility levels in various regions of the ALR2 protein, which were calculated. The C-α backbones RMSF for ALR2-Apo and ALR2-**8b** showed defined peaks at 218–228, but ALR2-**6b** and ALR2-**7b** displayed more flexible residues, especially 120–140 and 301–318 (loops covering and proximal to the active site). Interestingly, the RMSF findings (Figure 7B) corroborate the RSMD values. Moreover, the average RMSF values for ALR2-Apo, ALR2-**8b**, ALR2-**6b**, and ALR2-**7b** were 0.78 Å, 0.71 Å, 0.67 Å, and 0.62 Å, respectively.

**Figure 8:**
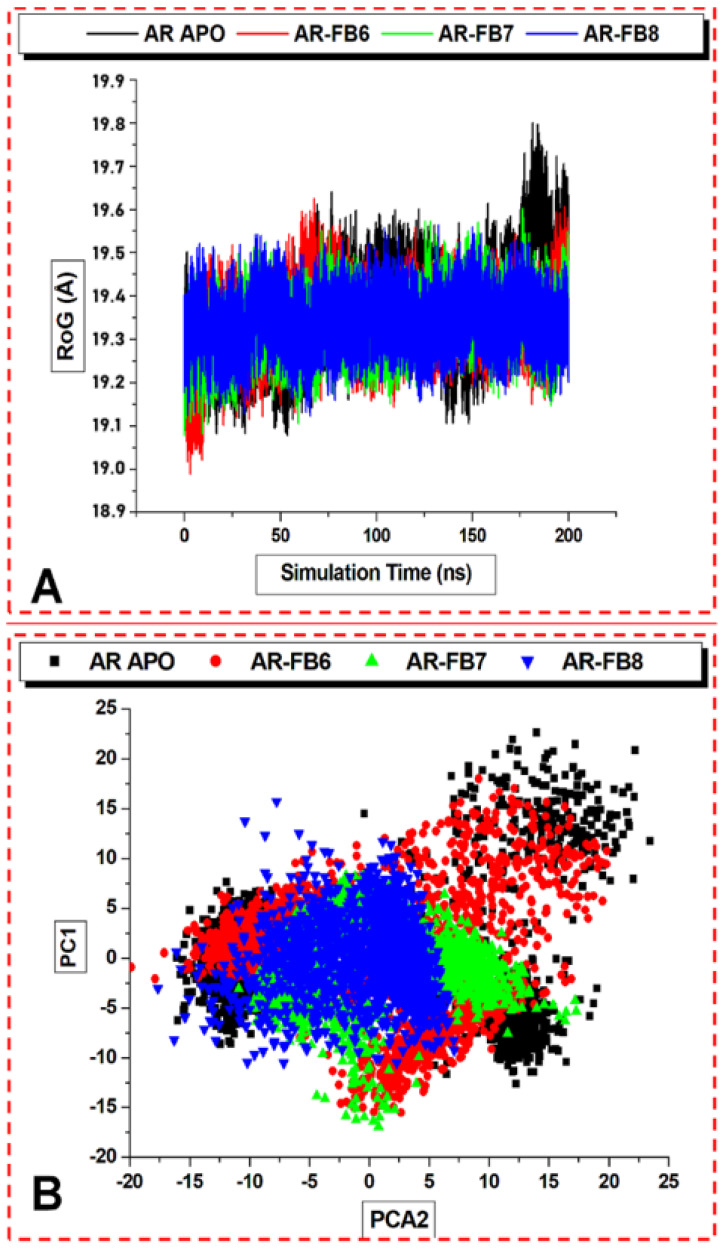
The radius of gyration, RoG (**A**), and the principal component analysis, PCA (**B**), illustrate the compactness and displaced inter-atoms of the ALR2 protein, respectively.

The C-α radius of gyration (RoG) analysis measures the compactness of a system and further provides an understanding of the perturbations occurring in the ALR2 protein. A C-α atom of proteins having the highest radius of gyration for the system size range suggests less tight packing proteins and vice versa. Figure 8A showed the RoG values of the inhibitor-bound ALR2 systems over 200 ns simulations decreased insignificantly at a pressure of 1 bar compared to the unbound protein. The average C-α atoms RoG of systems Apo-ALR2, ALR2-**8b**, ALR2-**6b**, and ALR2-**7b** were 19.36, 19.33, 19.33, 19.33, respectively. There was no difference in the RoG of complexes. Therefore, the Apo system seems less compact than the bound systems.

Furthermore, the interactions and mechanisms of inhibition for the ligands were investigated by computing the principal component analysis (PCA). The analysis helps identify the most strongly correlated variables with each component or the numbers with the large magnitude farthest from zero in either direction [46]. The level at which a correlation is essential is determined, for instance, a correlation above a given value may be deemed necessary. PCA reveals the displacement of the ALR2 C-α atoms during MD simulations performed using the CPPTRAJ in AMBER18 GPU by computing the first two components, PC1 and PC2. The MD simulations’ convergence in the space was obtained from the averaged structure covariance matrix using Cartesian coordinates. PC1 and PC2 were plotted along the vertical and horizontal axes, respectively, which indicate covariance matrix after eigenvector elimination. Each point between the single-directional motions was uniquely orientated due to the overlapping of the typical structural conformations. Figure 8B illustrates the scatter plot generated by the unbound protein (black), ALR2-**8b** (red), ALR2-**6b** (green), and ALR2-**7b** (blue). The plot showed significant differences among the systems, indicating variations in the overall motion between the unbound system and the complexes. The critical protein motions along specific directions were represented by eigenvectors, given by the normal modes.

**Figure 9:**
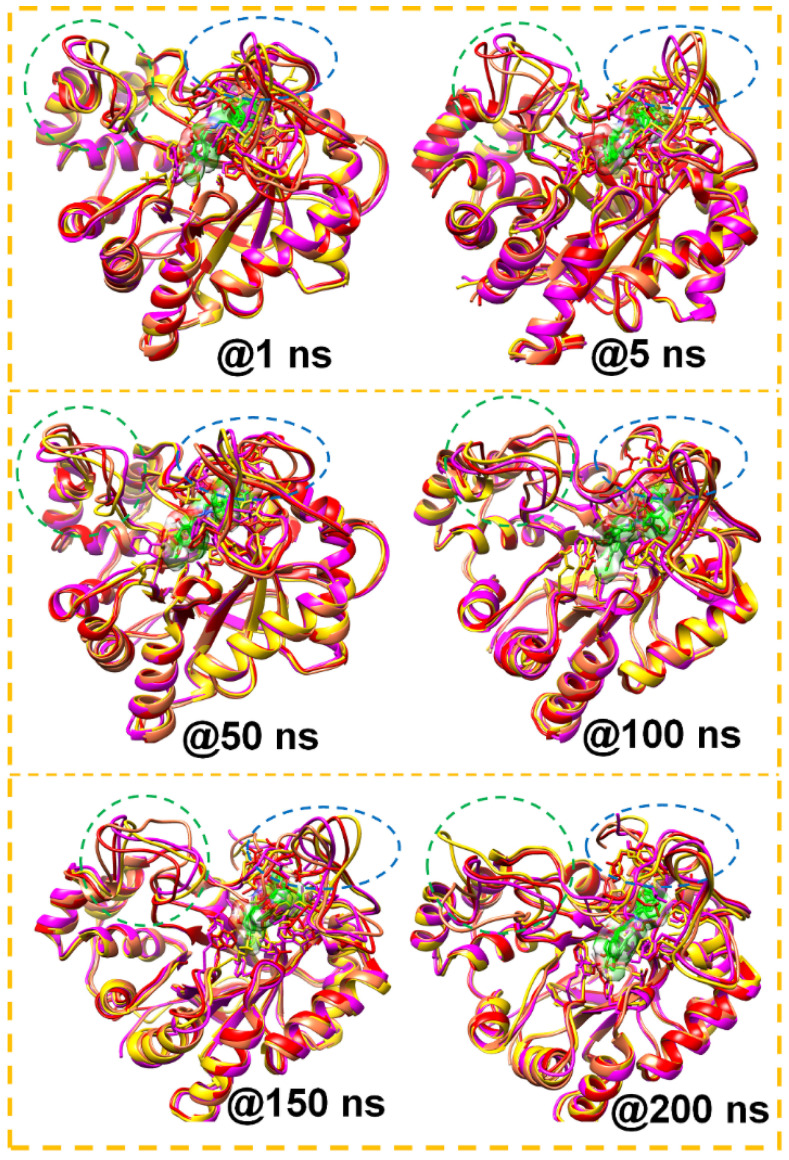
The MD simulation snapshots showed the loop structures at the time interval (ns) 1, 5, 50, 100, 150, and 200. Residues in the 218–228 region are enclosed in green and those in the 300–318 region are enclosed in blue circles.

Further investigation into the inhibition actions of the **6b**, **7b**, and **8b** against ALR2 revealed observation of significant visual structural changes in the loops proximal to and covering the pocket of the enzyme. The study of loops provides insights into various functions of proteins, including binding properties, dynamics, and shape [62]. Loop structures consist of 218–228 and 300–318 residues. We can refer to these residue regions as loops A and B. The trajectory snapshots were collected in nanoseconds at simulation time intervals (1, 5, 50, 100, 150, and 200). Figure 9 reveals the differential changes in the loop structures of the Apo, ALR2-**6b**, ALR2-**7b**, and ALR2-**8b** systems.

Interestingly, loop A snapshots showed that loop A moved to cover the ALR2 active site, especially in the ALR2-**8b** complex as the simulation progressed.

On the other hand, loop B displayed twisting behavior throughout the 200 ns simulations. Such structural changes are critical for the functional activity of proteins, including aldose reductase. For instance, the opening and closing of loop A could impact the ligand-binding terrain of the ALR2 protein to enhance the binding affinity of the inhibitors.

**Figure 10:**
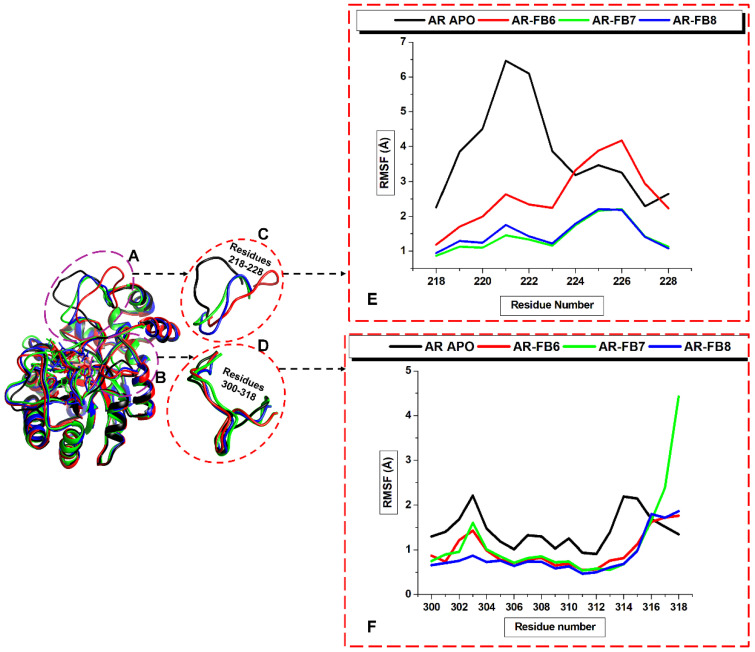
Loop dynamics plot for residues 218–228 (**A**) and 300–318 (**B**). Zoomed-out snapshots of the loops A (**C**) and loop B (**D**). RMSF of the superimposed Apo and the bound ALR2 systems to show loop A (**E**) and loop B (**F**).

Therefore, we further investigated the dynamics of these two loops around the ALR2 active site over 200 ns MD simulations by computing the RMSF for the loop structures to ascertain the observed visual changes. Figure 10 shows the RMSF plots of loops A and B. Of course, the loops RMSF showed fluctuations. The fluctuations displayed by the Apo and ALR2-**8b** are significant, while ALR2-**6b** and ALR2-**7b** changes were almost the same. Indeed, these dynamic loop analyses further confirm the ligands’ inhibition of ALR2 activity.

**Figure 11:**
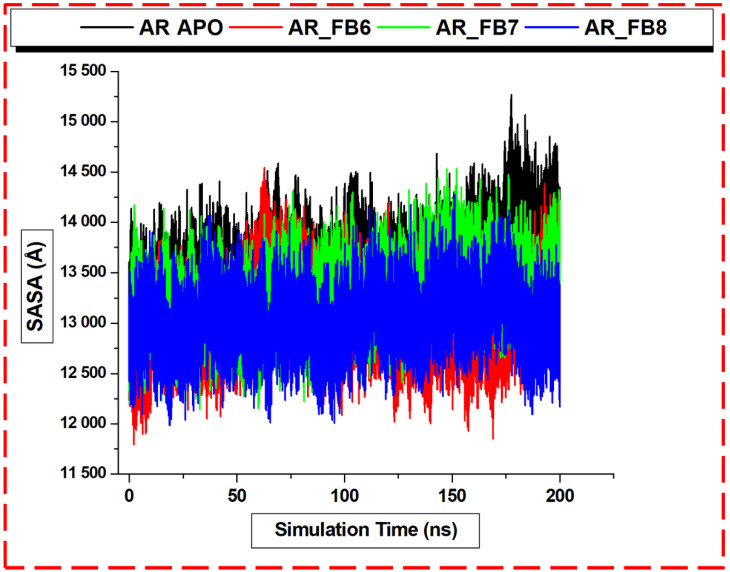
Solvent accessible surface area (SASA) plots of unbound protein (black, working code AR–Apo), and ALR2-**8b** (red, working code AR-FB6), ALR2-**6b** (green, working code AR-FB7), and ALR2-**7b** (blue, working code AR-FB8) systems.

The molecular dynamic (MD) simulations technique and solvent accessible surface area (SASA) have been explored to study proteins’ and solvents’ conformational movements [63]. The comparison of the MD simulations at low and high pressure gives rise to microscopic pressure-induced denaturation, findings that corroborate experimental data. We performed 200 ns simulations to observe the unfolding conformational events in the Apo and inhibitor-bound systems by investigating the SASA. The SASA analysis of ALR2 systems’ dynamics and motions further corroborates the above findings. The average SASA (Å) values for the Apo, ALR2-**8b**, ALR2-**6b**, and ALR2-**7b** were 13,552.9, 13,054.3, 12,277.8, and 13,037.6, respectively. The SASA difference of the unbound and bound with the highest SASA value was about 500 Å. These results (Figure 11) revealed significantly reduced solvent values in the bound systems, with ALR2-**6b** having the lowest SASA. The inhibitors induced reorientation of the proximal residues to the active site and residues that make up the site. The more the time evolution of simulation and the more burying of the inhibitors, the more likely the solvent reduction was at the protein–ligand interface. Moreover, the SASA trajectories of aldose reductase for polar residues include charged groups, non-polar species, and whole protein. Hence, we could infer that the hydrophobic residues in ALR2 surfaces are more exposed than the polar or charged residues, accounting for the decrease in the solvent accessibility of the bound ALR2 systems.

## 4. Conclusions

This work described a novel series of (*E*)-benzaldehyde *O*-(benzyl)-oximes as ALR2 inhibitors possessing ancillary anti-oxidant properties. Inspired by natural compounds, they are characterized by an (*E*)-benzaldehyde O-benzyl oxime core structure, which has already proven to be a key fragment for the obtainment of effective enzymatic inhibitors [64,65]. A comprehensive polyhydroxy substitution pattern on the benzaldehyde residue has been investigated, in a combination with suitable substituents on the benzyl moiety. As for the ALR2 inhibitory efficacy, the 2,3,4-trihydroxy substituents turned out to be the most promising, once combined with a 3- or 4-methoxy group on the benzyl ring. In fact, compound (*E*)-2,3,4-trihydroxybenzaldehyde *O*-(3-methoxybenzyl) oxime (**7b**) and (*E*)-2,3,4-trihydroxybenzaldehyde *O*-(4-methoxybenzyl) oxime (**8b**) displayed favorable ALR2 inhibitory activity, corroborated by both molecular docking and molecular dynamics studies. The ligand-bound ALR2 systems showed insignificant average RMSD and RMSF, implying relatively stable simulations and thoroughly equilibrated bound systems. The PRED analysis revealed the detailed residue energy contributions for each complex. As for the anti-oxidant efficacy, both the 2,3,4-trihydroxy and the 3,4-dihydroxy substitution patterns conferred a remarkable activity, which proved to be well above the one shown by natural compounds such as resveratrol and pterostilbene, used as the reference compounds. Results obtained confirm the relevance of hydroxy groups to give anti-oxidant properties to heterocyclic compounds, as already demonstrated by several authors for similar scaffolds [66,67]. Although far from being conclusive, results obtained with the novel series demonstrated the usefulness of the proposed scaffold for the obtainment of active compounds, suggesting the (*E*)-benzaldehyde *O*-(benzyl)-oximes as prototypes of novel anti-oxidant ARIs deserving to be further investigated.

## Figures and Tables

**Table 1:**
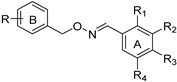
Substitution pattern of (*E*)-benzaldehyde *O*-benzyl oximes **6a–e**, **7a–e**, **8a–e**, and **9–11**.

Compound	R	R_1_	R_2_	R_3_	R_4_
**6a**	2-OCH_3_	H	OH	H	OH
**6b**	2-OCH_3_	OH	OH	OH	H
**6c**	2-OCH_3_	H	OH	OH	H
**6d**	2-OCH_3_	OH	H	H	OH
**6e**	2-OCH_3_	OH	H	OH	H
**7a**	3-OCH_3_	H	OH	H	OH
**7b**	3-OCH_3_	OH	OH	OH	H
**7c**	3-OCH_3_	H	OH	OH	H
**7d**	3-OCH_3_	OH	H	H	OH
**7e**	3-OCH_3_	OH	H	OH	H
**8a**	4-OCH_3_	H	OH	H	OH
**8b**	4-OCH_3_	OH	OH	OH	H
**8c**	4-OCH_3_	H	OH	OH	H
**8d**	4-OCH_3_	OH	H	H	OH
**8e**	4-OCH_3_	OH	H	OH	H
**9**	2-Cl	H	OH	H	OH
**10**	3-Cl	H	OH	H	OH
**11**	4-Cl	H	OH	H	OH

**Table 2:** ALR2 inhibitory activities of (*E*)-benzaldehyde *O*-benzyl oximes **6a–e**, **7a–e**, **8a–e**, and **9–11**.

Compound	% Inhibition ^a^	Compound	% Inhibition ^a^
**6a**	n.a. ^b^	**8a**	n.a.
**6b**	44	**8b**	67
**6c**	19	**8c**	25
**6d**	28	**8d**	n.a.
**6e**	23	**8e**	22
**7a**	n.a.	**9**	10
**7b**	63	**10**	44
**7c**	20	**11**	n.a.
**7d**	n.a.		
**7e**	23		

^a^ Percentage of enzyme inhibition at 100 μM test compounds, obtained as mean of at least three determinations. Standard errors of the means (SEMs) are ≤10%. ^b^ Not active. No inhibition was observed at 100 μM test compound.

**Table 3:** Binding energies obtained from the molecular docking of compounds **6b**, **7b**, and **8b** into different conformations of human ALR2.

	Binding Energies (kcal/mol)		
PDB Code	ALR2-6b	ALR2-7b	ALR2-8b
2F2B	−4.9	−7.6	−4.8
4JIR	−7.3	−8.1	−7.8
4IGS	−8.0	−8.4	−8.0
4JII	−9.0	−9.4	−8.2

**Table 4:** Thermodynamics analysis. Summary of MM/GBSA-based binding free energy contributions to the ALR2-**6b**, ALR2-**7b**, and ALR2-**8b** complexes.

Complex	Energy Components (kcal/mol)						
	ΔE_vdW_	ΔE_elec_	ΔG_gas_	EG_GB_	E_SA_	ΔG_solv_	ΔG_bind_
ALR2-**6b**	−39.29 (±3.22)	−11.81 (±6.19)	−51.10 (±7.66)	30.94 (±4.71)	−5.24 (±0.23)	25.70 (±4.69)	−25.40 (±4.66)
ALR2-**7b**	−40.99 (±3.93)	−21.88 (±6.55)	−62.88 (±6.65)	33.87 (±3.42)	−5.50 (±0.37)	28.38 (±3.30)	−34.51 (±4.81)
ALR2-**8b**	−42.22 (±1.73)	−7.50 (±4.27)	−49.72 (±4.63)	23.89 (±2.76)	−5.65 (±0.15)	18.24 (±2.76)	−31.48 (±2.92)

ΔE_elec_ (electrostatic), ΔE_vdw_ (van der Waals), ΔG_bind_ (calculated total free binding energy) ΔG_gas_ (gas-phase energy) and ΔG_solv_ (solvation free energy).

## Data Availability

Not applicable.

## Supplementary Materials

The following supporting information can be downloaded at: https://www.mdpi.com/article/10.3390/biom12030448/s1. Figure S1: Purification of ALR2 by size-exclusion chromatography (SEC); Figure S2: LCMS + MALDI Characterization of ALR2; Figure S3: Structures of ALR2 conformation, showing some active site residues (A) and chemical structures of inhibitors **6b**, **7b**, and **8b**; Table S1: IR spectral signatures of the synthesized compounds **6a–e**, **7a–e**, **8a–e,** and **9–11**; Figure S4: Effects of (*E*)-benzaldehyde *O*-benzyl oximes **6a****–e**, **7a****–e**, **8a****–e**, and **9****–11**, and Resveratrol on the production of thiobarbituric reactive substances (TBARS) in rat brain homogenate; Figure S5: ALR2-**6b** molecular docked pose; Figure S6: ALR2-**8b** molecular docked pose; Figure S7: ALR2-**7b** molecular docked pose.
