# Supplementary material for: Nature-Inspired O-Benzyl Oxime-Based Derivatives as New Dual-Acting Agents Targeting Aldose Reductase and Oxidative Stress
Source: Biomolecules. 2022 Mar 14;12(3):448. doi: 10.3390/biom12030448 (PMC8946157; doi:10.3390/biom12030448)
Supplement: Supplementary file 1 [file biomolecules-12-00448-s001.zip › biomolecules-1596598-supplementary.pdf]

Supplementary Materials

Nature inspired *O*-benzyl Oxime-based derivatives as new dual acting agents targeting Aldose reductase and oxidative stress

Lidia Ciccone,[1,2,3] Giovanni Petrarolo,[1] Francesca Barsuglia,[1] Carole Fruchart-Gaillard,[2] Evelyne Cassar Lajeunesse,[2] Adeniyi T. Adewumi,[4] Mahmoud E. S. Soliman,[4] Concettina La Motta,[1,3,*] Elisabetta Orlandini,[3,5,6] Susanna Nencetti.[1,3,*]

1 Department of Pharmacy, University of Pisa, Via Bonanno 6, 56126 Pisa, Italy.
2 Département Médicaments et Technologies pour la Santé (DMTS), Université Paris Saclay, Commissariat à l'Énergie Atomique et aux Énergies Alternatives (CEA), Institut National de Recherche pour l'Agricolture, l'Alimentation et l'Environment (INRAE), SIMoS, 91191 Gif-sur-Yvette, France.
3 Centre for Instrumentation Sharing, University of Pisa (CISUP), Lungarno Pacinotti 43, 56126 Pisa, Italy.
4 Molecular Bio-Computation and Drug Design Laboratory, School of Health Science, University of KwaZulu-Natal, Westville Campus, 4001 Durban, South Africa.
5 Department of Earth Sciences, University of Pisa, via Santa Maria 53, 56126 Pisa, Italy.
6 Research Center "E. Piaggio", University of Pisa, Largo Lucio Lazzarino 1, 56122 Pisa, Italy.

Table of Contents

Figure S1. Purification of ALR2 by Size-exclusion Chromatography (SEC).

Figure S2. LCMS+MALDI Characterization of ALR2.

Figure S3. Structures of ALR2 conformation, showing some active site residues (A) and chemical structures of inhibitors **6b** (B), **8b** (C), and **7b** (D).

Figure S4. Effects of (*E*)-benzaldehyde *O*-benzyl oximes **6a-e**, **7a-e**, **8a-e**, and **9-11**, and Resveratrol on the production of Thiobarbituric Reactive Substances (TBARS) in rat brain homogenate.

Figure S5. ALR2-**6b** molecular docked pose.

Figure S6. ALR2-**8b** molecular docked pose.

Figure S7. ALR2-**7b** molecular docked pose.

Table S1. List of IR spectral signatures of the synthesized compounds **6a-e**, **7a-e**, **8a-e** and **9-11.**

Figure S1. Purification of ALR2 by Size-exclusion chromatography (*SEC*) Sephacryl® S-100 HR GE Healthcare.

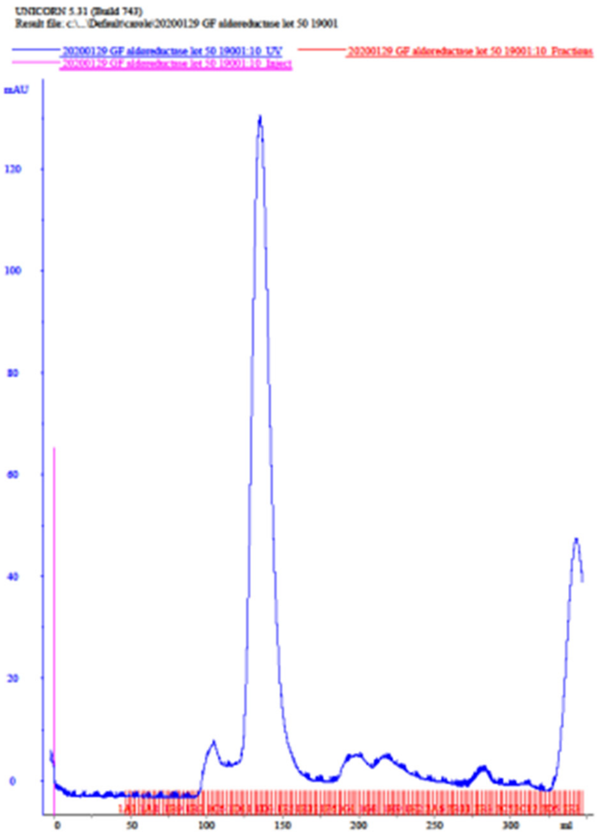

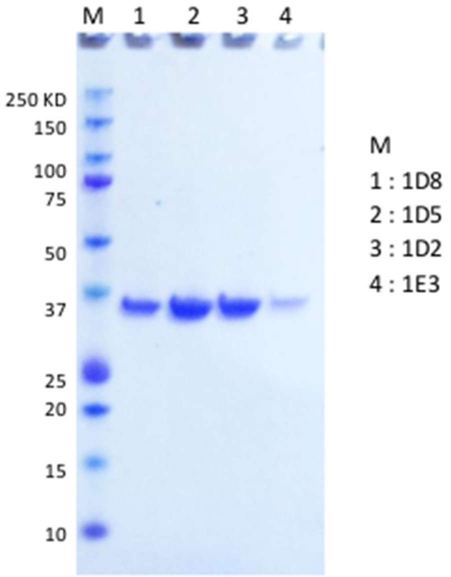

Figure S2. LCMS+MALDI of ALR2 after purification by SEC Sephacryl; m/z: 35909.1 +/- 0.9 Da.

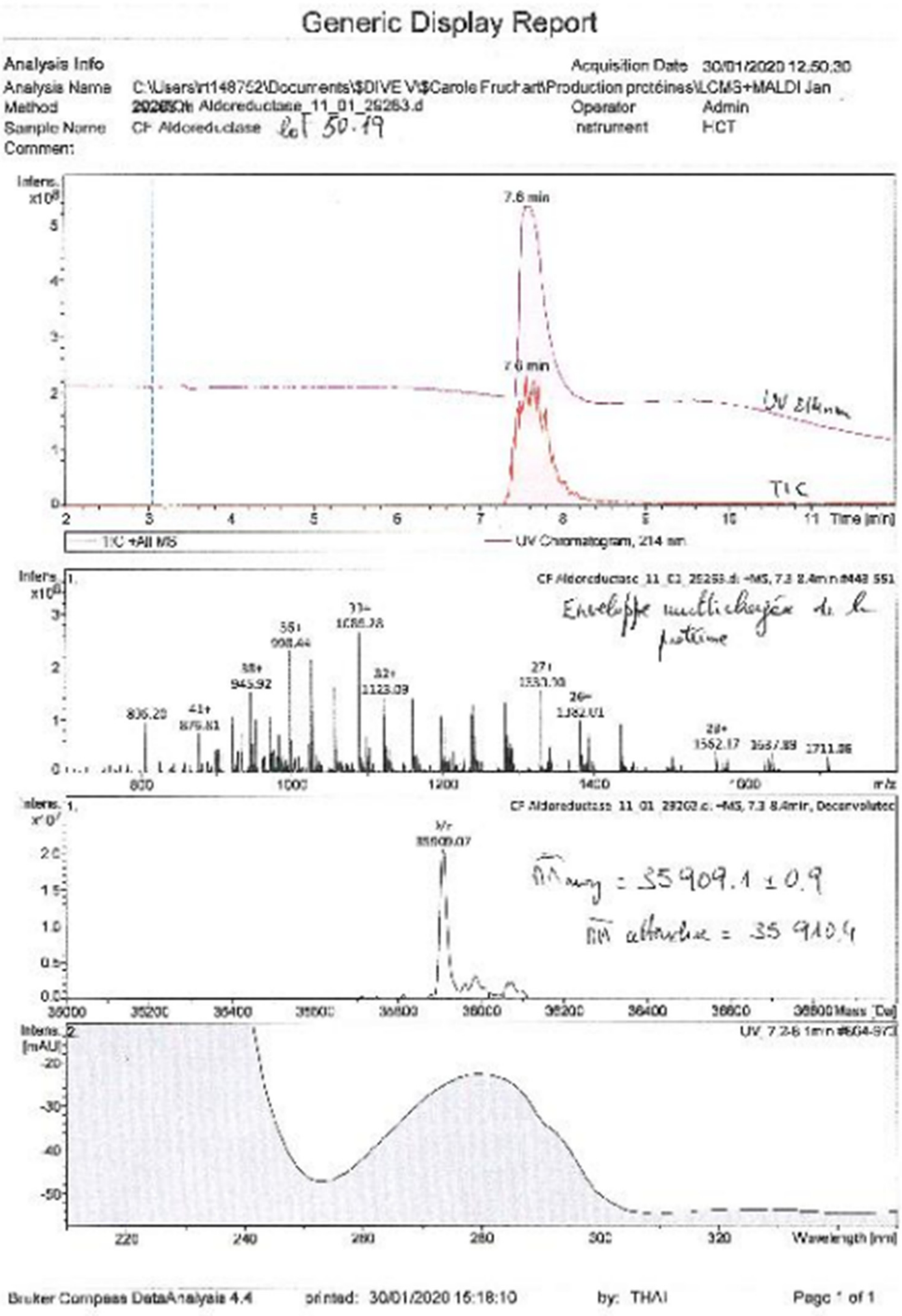

Figure S3. Structures of human recombinant aldose reductase (ALR2) showing some active site residues (A), and the newly proposed inhibitors (*E*)-benzaldehyde *O*-benzyl oximes **6b** (B, working code FB7), **8b** (C, working code FB6), and **7b** (D, working code FB8).

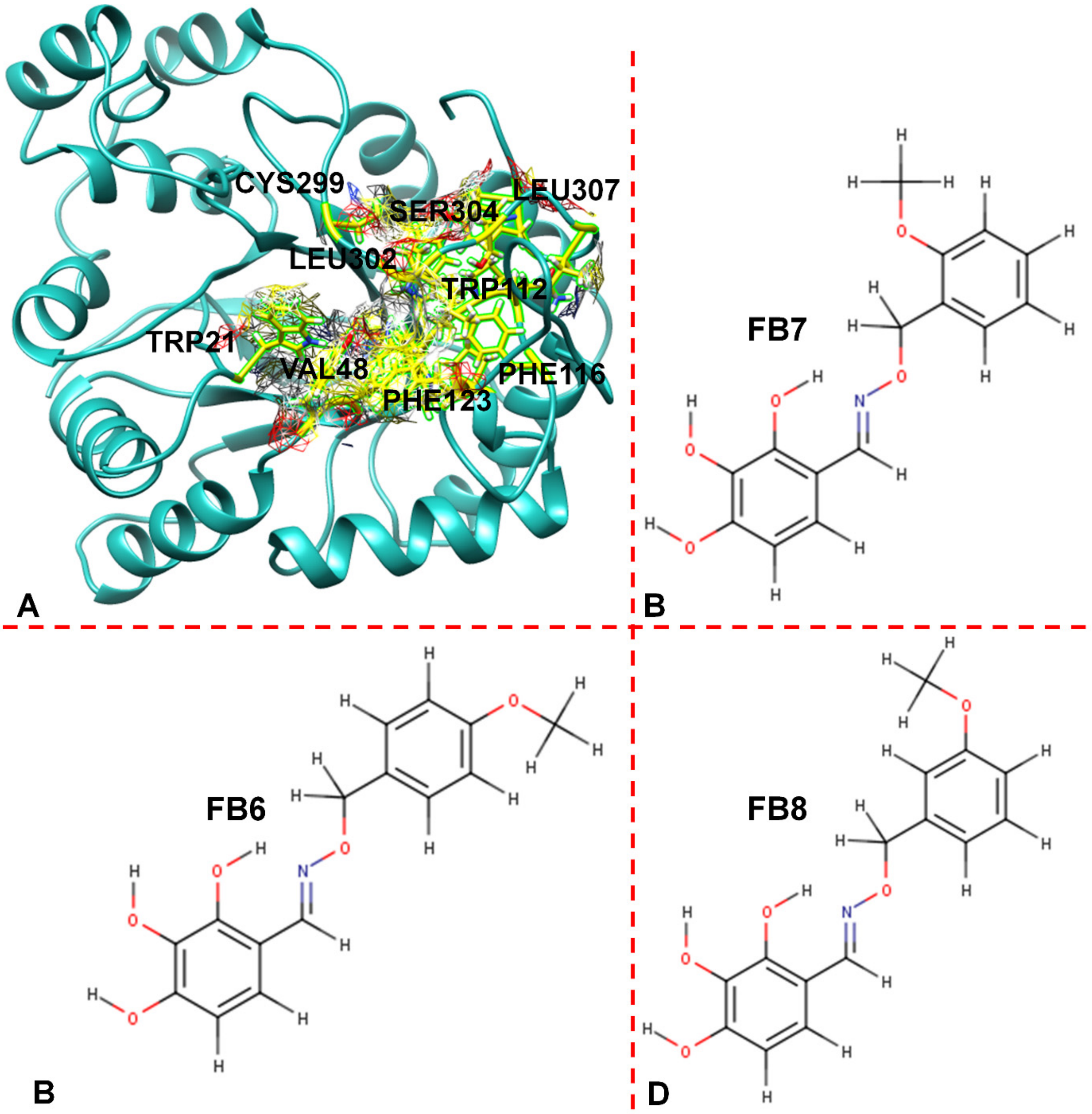

Figure S4. Effects of (*E*)-benzaldehyde O-benzyl oximes **6a-e**, **7a-e**, **8a-e**, and **9-11**, and Resveratrol, tested at 1 μM, on the production of Thiobarbituric Reactive Substances (TBARS) in rat brain homogenate.

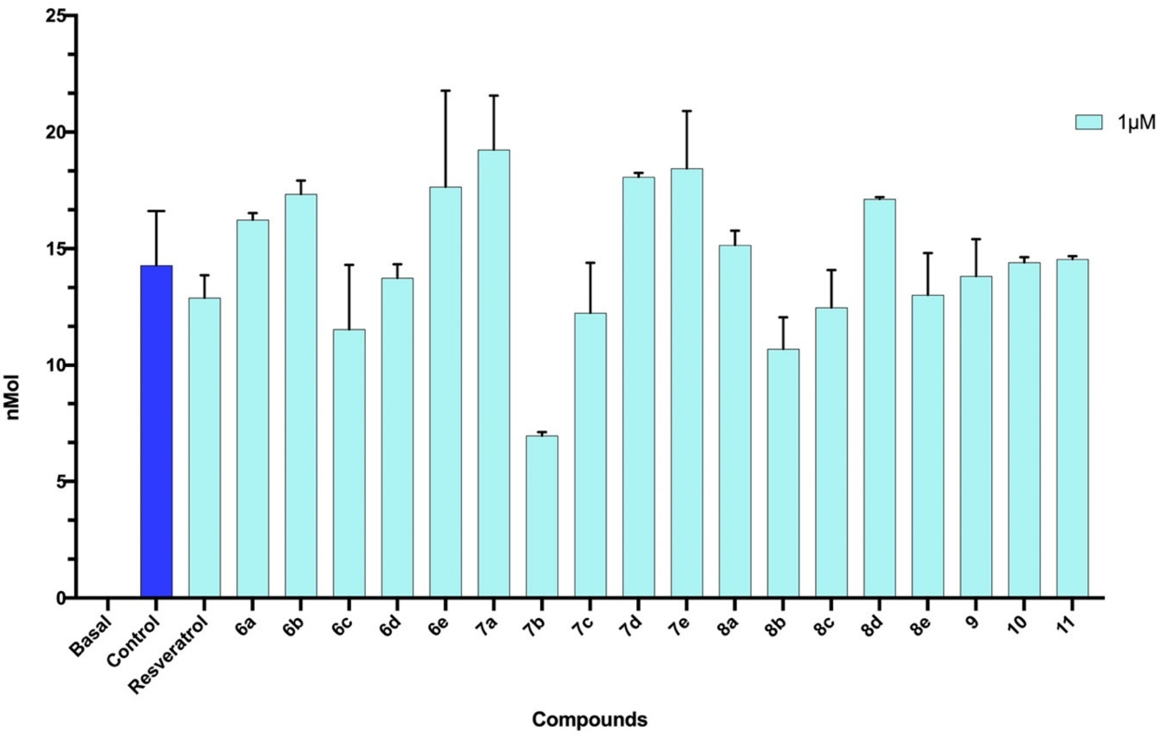

Figure S5. ALR2-**6b** molecular docked pose. (A) Docked pose showing the ALR2 (pink) housed **6b** (green, working code FB7)) and NADP+ (brown). (B) Interaction types include π – π T-shaped, π – π stacked, π – sigma and π - alkyl hydrophobic interactions.

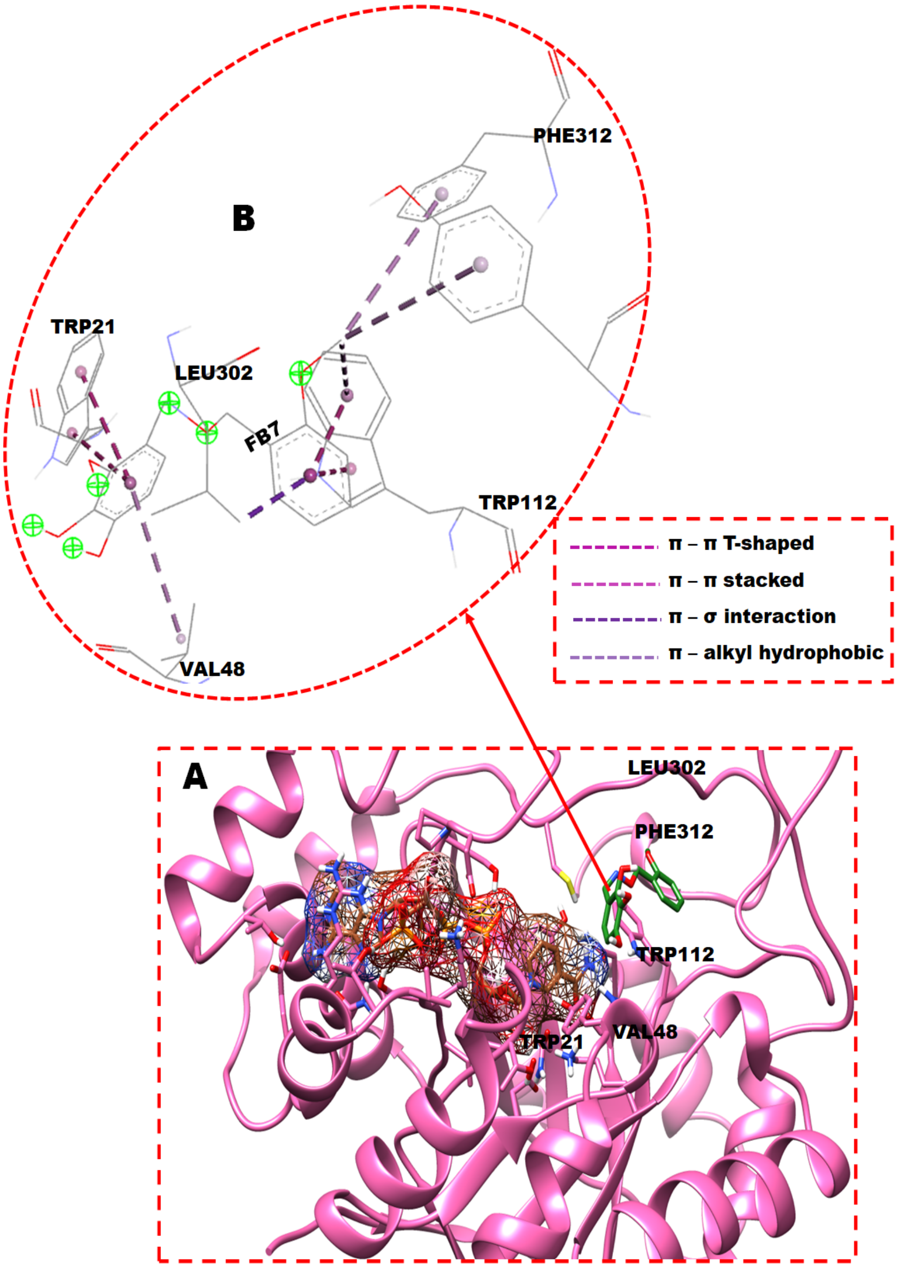

Figure S6. ALR2-**8b** molecular docked pose. (A) Docked pose showing the ALR2 (pink) housed **8b** (coral, working code FB6) and NADP+ (brown). (B) Interaction types include H-bonds, π – π T-shaped, π – π stacked, π – sigma and π - alkyl hydrophobic interactions.

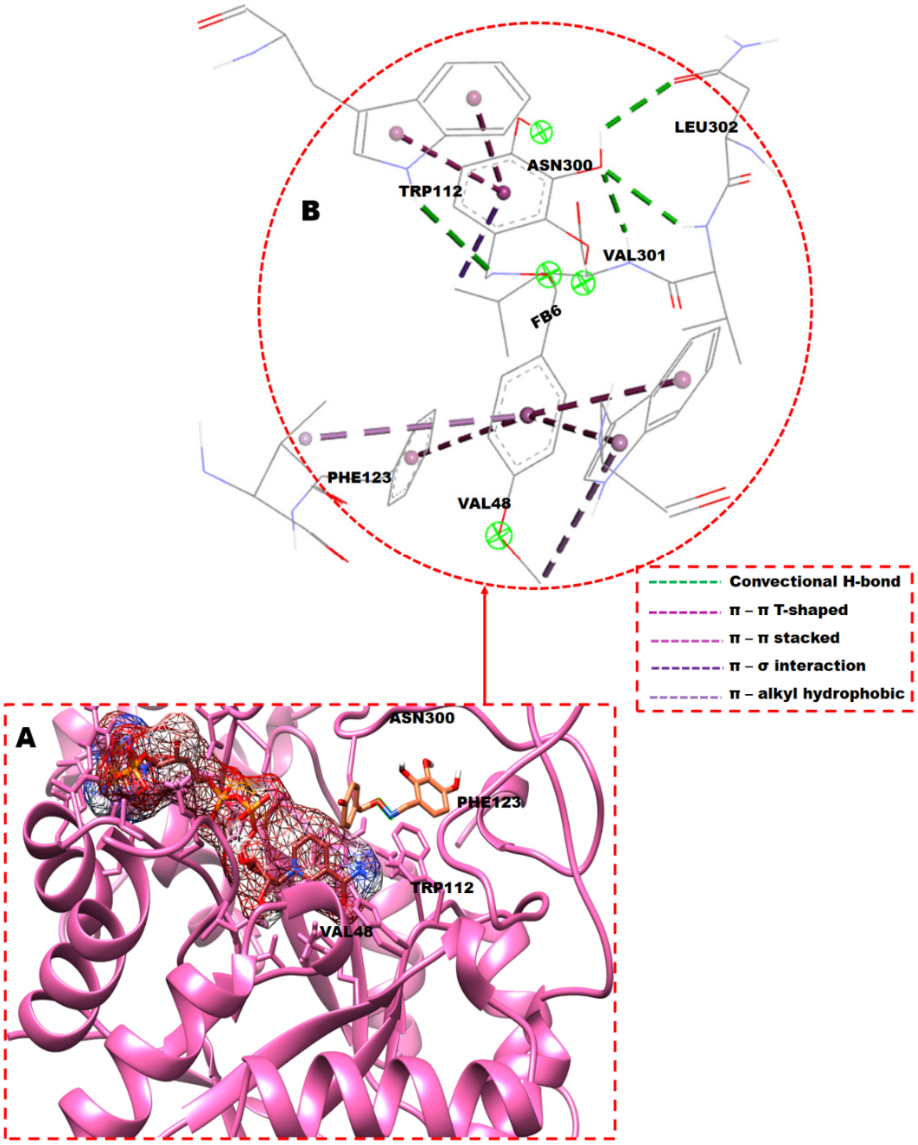

Figure S7. ALR2-**7b** molecular docked pose. (A) Docked pose showing the ALR2 (pink) housed **7b** (yellow, working code FB8) and NADP⁺ (brown). (B) Interaction types include H-bonds, π – π T-shaped, π – π stacked, π – sigma and π – alkyl, sulphur, and alkyl hydrophobic interactions.

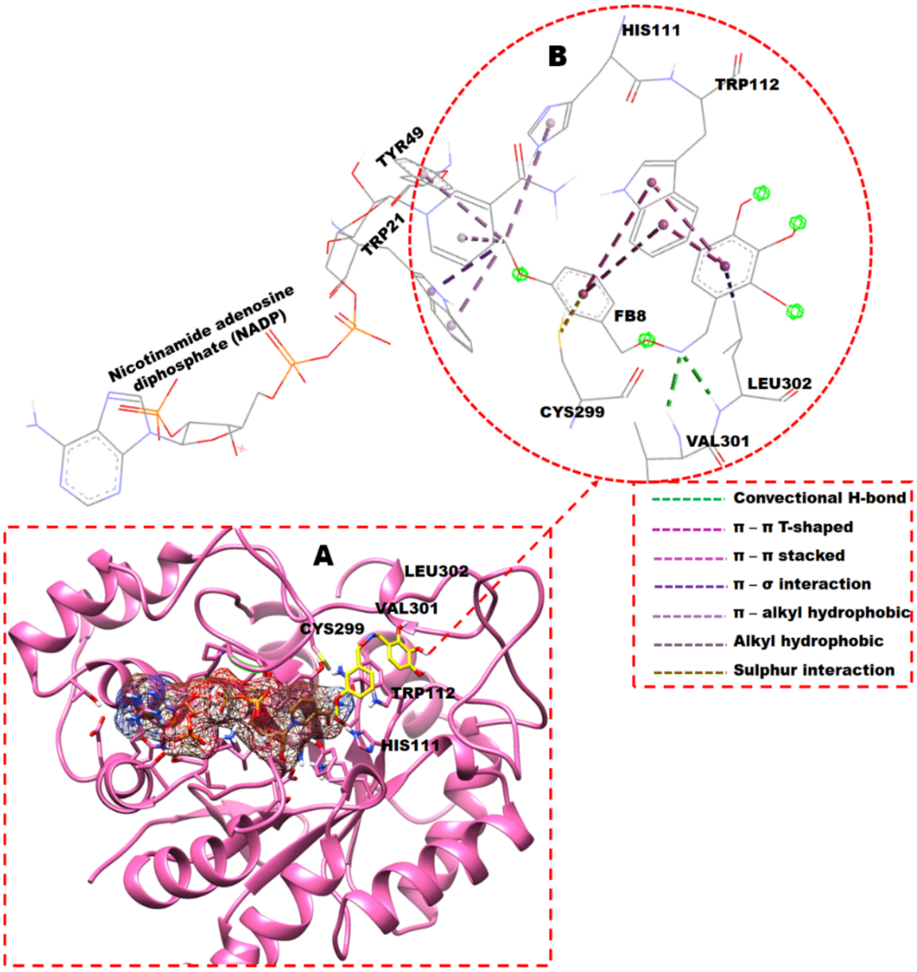

Table S1. List of IR spectral signatures of the synthesized compounds **6a-e**, **7a-e**, **8a-e** and **9-11**.

Vibration type	Wavelengths (cm^{-1})	Comments
-O-H stretching	3500-3200	
-C-H stretching	2880-2980	
-C=N- stretching	1600-1630	
-N-O- stretching	1584-1596	
H_3C-O- stretching	1234-1266	Absent in spectra of compounds **9-11**
-O-H bending	1295-1367	
-C-O- stretching	1279-1322<br>1009-1082	Aromatic C-O
-C-O- stretching	1147-1199	-C-O-N-
-C-H bending	942-999	Aromatic C-H and N=C-H
C-Cl stretching	800.4, *para*<br>779.0, *meta*<br>753.3, *ortho*	Spectra compound **9-11**
